# Inhibition of autophagy enhances the antitumor efficacy of T/CAR T cell against neuroblastoma

**DOI:** 10.1186/s13046-025-03453-0

**Published:** 2025-07-03

**Authors:** Francesca De Mitri, Manuela Giansanti, Ombretta Melaiu, Dorothee Haas, Stefan Ebert, Nicola Tumino, Elisabetta Vulpis, Francesca Gatto, Beatrice Martuscelli, Manuela Antonioli, Elisabetta Sangiuliano, Simona Caruso, Marco Scarsella, Cristiano De Stefanis, Veronica Marabitti, Silvia Campello, Doriana Fruci, Paola Vacca, Ignazio Caruana, Francesca Nazio

**Affiliations:** 1 Department of Biology, University of Rome Tor Vergata, Rome, Italy; 2 Innate Lymphoid Cells Unit, Bambino Gesù Children’s Hospital IRCCS, Rome, Italy; 3 Department of Pediatric Hematology and Oncology of Cell and Gene Therapy, Bambino Gesù Children’s Hospital IRCCS, Rome, Italy; 4 Department of Clinical Sciences and Translational Medicine, University of Rome “Tor Vergata”, Rome, Italy; 5 Department of Pediatrics - Hematology, Oncology and Stem Cell Transplantation Unit, University Hospital Würzburg, Würzburg, Germany; 6 Department of Laboratory Medicine, Division of Pathology, Karolinska Institute, Huddinge, Sweden; 7 National Institute for Infectious Diseases, “Lazzaro Spallanzani” - IRCCS, Rome, Italy; 8 Research Laboratories, Bambino Gesù Children’s Hospital IRCCS, Rome, Italy

**Keywords:** Neuroblastoma, Immune cells, GD2.CAR T, Chloroquine, ULK1 inhibitor

## Abstract

Neuroblastoma (NB) is the most common extracranial solid tumor in children characterized by poor immune infiltration and resistance to adaptive immunity, contributing to its limited response to immunotherapy. A key mechanism underlying immune evasion in cancer is autophagy, a cellular process that plays many roles in cancer by supporting tumor survival and regulating immune interactions. In this study, we investigate the impact of autophagy inhibition on NB tumor growth, immune modulation, and the efficacy of immunotherapy. Using both murine and human NB cell lines, we demonstrate that genetic and pharmacological inhibition of autophagy significantly reduces 3D spheroid growth and upregulates major histocompatibility complex class I (MHC-I) expression. In vivo studies further confirm that targeting autophagy suppresses tumor progression and promotes immune infiltration into the tumor. Notably, we observe a significant increase in CD8^+^ T cell recruitment and activation, suggesting that autophagy inhibition reshapes the immune landscape of NB, rendering it more susceptible to immune-mediated clearance. Crucially, autophagy inhibition also sensitizes NB cells to T cell-mediated cytotoxicity and enhances the therapeutic efficacy of GD2.CAR T-cell therapy. In vitro co-culture assays reveal increased CAR T cell-mediated tumor killing upon autophagy blockade, while in vivo models show prolonged tumor control and improved survival in treated mice compared to CAR T-cell therapy alone. These findings highlight autophagy as a key regulator of immune evasion in NB and suggest that its inhibition could serve as a promising therapeutic strategy to enhance immune recognition and improve the efficacy of immunotherapy.

**Supplementary Information:**

The online version contains supplementary material available at 10.1186/s13046-025-03453-0.

## Introduction

Neuroblastoma (NB) is the most common extracranial solid tumor in children arising from primitive neural crest cells of the developing sympathetic nervous system (SNS) [1, 2]. It is responsible for 15% of childhood cancer deaths. Several prognostic factors such as age at diagnosis, DNA index (ploidy), MYCN amplification, and tumor stage are used to stratify NB into very low-risk, low-risk, intermediate-risk, and high-risk (HR) groups [3]. Current standard therapies for HR patients include chemotherapy, radiation, and surgery [4]. However, these therapies induce long-term toxicities and often provide limited benefit, thus driving efforts to exploit the immune system to counteract NB aggressiveness. HR-NB, indeed, are classified as immunologically “cold” due to limited T cell infiltration, low expression of HLA-Class I major histocompatibility complex class I (MHC-I) molecules, and low tumor mutational burden leading to an immunosuppressive tumor microenvironment (TME) [5–9]. In NB, low surface expression of MHC-I molecules is regulated by epigenetic and/or post-transcriptional mechanisms [10, 11]. This reduced expression can be reversed by treatment with inflammatory cytokines such as IFNγ and TNFα [10–13]. Moreover, TME of NB is poorly immunogenic. Several pre-clinical and clinical studies have highlighted the importance of adequate and sustained T infiltrate lymphocyte activity to achieve NB eradication or at least tumor control. In the context of NB, immunotherapy appears to be a promising strategy, as evidenced by recent positive outcomes with the antibody (Ab) dinutuximab β [14] as well as with Chimeric Antigen Receptor (CAR) T-cell therapy targeting GD2 [15]. While these findings are encouraging, further investigation is needed to overcome intrinsic limitations to CAR T cell efficacy in solid tumors. Specifically, key challenges include inadequate CAR T cell penetration and survival within the TME. To this aim, an attractive strategy could be to convert “cold tumors” to “hot tumors”, which may promote effective antitumor response and increase the performance of immunomodulatory therapies. One of the mechanisms that maintain stability within the components of the TME shaping its immunosuppressive nature is autophagy. Autophagy is a self-degradative process, essential for the maintenance of metabolic and genetic homeostasis in all eukaryotic organisms. By autophagy, cellular components are delivered to lysosomes to ensure the basal turnover of cytosolic organelles and provide energy and macromolecular precursors. Autophagy impairment or its excessive activity has been associated with several human disorders, ranging from cancer to autoimmunity and neurodegenerative disease [16]. In cancer, autophagy is a Janus-faced process, acting both as a tumor suppressor (cleaning cells from damaged organelles) and as a tumor adaptive response (favoring malignant progression and chemoresistance).

Several preclinical studies on adult cancers identified autophagy as a key regulator of immune responses [17]. Autophagy has been reported to influence homeostasis, survival, activation, proliferation, and differentiation of immune system components, such as natural killer (NK) cells, macrophages, dendritic cells, and T and B lymphocytes [18]. Altered autophagy in cancer, immune, or stromal cells can influence tumor-immune interactions, thereby reshaping the TME [19], affecting antigen release, antigen presentation, antigen recognition, and the trafficking of immune cells [20]. Meanwhile, autophagy also influences the release of cytokines and Abs. Also, MHC-I molecules present on the surface of Pancreatic Ductal Adenocarcinoma (PDAC) cells undergo degradation via autophagy [17]. Blocking autophagy can lead to the restoration of MHC-I levels on the surface of these cells [21, 22]. Moreover, the combined use of autophagy inhibitors and PD-1 or CTLA-4 immune checkpoint blocking monoclonal Abs (mAb) increases Cytotoxic T lymphocytes (CTL) infiltration in murine models of PDAC and non-small cell lung cancer (NSCLC), indicating a promising therapeutic approach for these tumors [23]. Other studies have shown that combining autophagy-targeted therapies with immunotherapy can enhance anti-tumor immune responses and improve clinical outcomes [24]. While these combination therapies hold potential, the exact mechanisms behind their effects are still under investigation.

In this work, by using both murine and human NB models in vitro and in vivo, we identified autophagy inhibition as a strategy to enhance the immunogenicity of NB cells. Specifically, we found that both genetic and pharmacological inhibition of autophagy can increase cell surface MHC-I expression and induce the recruitment of activated T cells into the TME. Furthermore, we speculatively proposed that autophagy might promote NB resistance to CAR-mediated cytotoxicity. We demonstrated that inhibition of autophagy dramatically sensitizes NB cells to GD2.CAR T cell-mediated killing both in vitro and in vivo. These results suggest that modulation of autophagy could represent a valuable approach to enhance the clinical efficacy of CAR T cell–based immunotherapy for NB, and may provide a rationale for the development of new therapeutic strategies targeting autophagy.

## Materials and methods

### Cell lines and culture conditions

Transgenic NB cell lines 9464D and 975A2 (provided by DF) derived from spontaneous tumors arising in TH-MYCN transgenic mice on a C57BL/6 background [25]. Murine NB cell lines (9464D and 975A2), human NB cell lines LAN5, SHEP and GI-LI-N (kindly provided by Dr. V. Pistoia, G. Gaslini Institute, Genova), and human leukemia THP-1 and HL-60 cell lines were growth in RPMI-1649 supplemented with 10% of Fetal Bovine Serum (FBS) and 1% of PenStrep and 2mM of glutamine; CHLA-255 and IMR32 were maintained in Iscove′s Modified Dulbecco′s Medium (IMDM) supplemented with 10% of FBS and 1% of PenStrep and 2 mM glutamine; SH-SY5Y were cultured in DMEM - F12 supplemented with 10% of FBS and 1% of PenStrep and 2 mM glutamine. Cells were grown in a humidified atmosphere at 37 °C and 5% CO2. Cells were routinely tested for the absence of mycoplasma.

### Treatments

Lyophilized SBI-0206965 (cat#29089, Cell Signaling Technology) was dissolved in DMSO to reach a 10 mM stock solution, and aliquots were stored at -20 °C. ULK-101 (cat#SML3480, Sigma-Aldrich) was dissolved in DMSO. Bafilomycin A1 (cat#B1793, Merck) powder was dissolved in DMSO at a stock concentration of 200µM, and aliquots were stored at -20 °C. For each experiment, an aliquot of drugs was thawed. Chloroquine powder (cat#C6628, Sigma-Aldrich) was dissolved in sterile water at 20 mM, and stored at 4 °C. Drugs were added to the wells the day after cell seeding. DMSO or sterile water was added in the control conditions.

### Spheroids generation, drug treatment, co-culture experiments, and analysis

Tumor spheroids were generated by seeding NB cell lines in ultra-low attachment 96-well plates (cat#7007, Corning Incorporated, USA) and centrifuged at 50 RCF for 1 min without braking and acceleration. Cell number seeded were 500 cells/well for murine NB cell lines, and 1000 cells/well for human NB cell lines. For experiments with genetic inhibition, shATG7 or shCTRL NB cells were seeded and after 72 h of spheroid generation, GD2.CAR T or non-transduced (NT) cells were added at different Effector:Target (E:T) ratios. For experiments with drug treatments, 72 h of neo-formed spheroids were treated with different concentrations of autophagy inhibitors (chloroquine or SBI-0206965). After 24–48 h of treatments, effector cells GD2.CAR T/NT cells were added at different E:T ratios. Spheroids growth and T cell cytotoxic activity were monitored at different time points using microplate multichannel automated imaging Celigo Image Cytometer (Nexcelom Bioscience) to assess changes in area and fluorescence intensity [26]. It is important to note that the average mean fluorescent intensity (MFI) represents the averages of the MFI of the four replicate spheroids.

In the spheroid viability assay, GI-LI-N cells were seeded, treated, and co-cultured with PRAME-TCR or NT as described above. To assess spheroids death and viability, calcein Green AM (cat#C34852, ThermoFisher Scientific) and propidium iodide (PI, cat#P4864, Sigma-Aldrich) were added in each well and incubated at 37 °C for 1h. The green and red fluorescence of calcein and PI were measured by Celigo Image Cytometer.

### Immunoblotting

Cells were lysed in the RIPA buffer (50 mM Tris–HCl pH 8.0/150 mM Sodium chloride/1% Np-40/0.5% sodium deoxycholate/0.1% sodium dodecyl sulfate/2 mM EDTA) containing 1× protease and 1× phosphatase inhibitor cocktails (Sigma-Aldrich). Protein samples were quantified using a BCA assay reagent in accordance with the manufacturer’s instructions. Protein samples were subjected to SDS-PAGE and transferred to polyvinylidene fluoride (PVDF) membranes in 25 mM Tris and 192 mM glycine. Membranes were overnight incubated with indicated antibodies at 4 °C. Following wash with PBS-T (PBS containing 0.1% Tween-20), the blot was incubated with corresponding peroxidase-labeled secondary Abs. Blots were developed with enhanced chemiluminescence (ECL, Millipore) reaction according to manufacturer instructions. The primary Abs used in this study were ATG7 (cat#8558, Cell Signaling), Actin (cat#A2066, Sigma), LC3 (cat#NB100-2220, Novus Biologicals), p62 (cat#PM045, MBL), p-ATG14 (cat#96752, Cell Signaling); ATG14 (cat#5504, Cell Signaling), ULK1 (cat#8054S, Cell Signaling).

### Cell viability assay

Cell proliferation was assessed by MTS (3-(4,5-dimethylthiazol-2-yl)-5-(3-carboxy- methoxyphenyl)-2-(4-sulfophenyl)-2 H-tetrazolium) assay after 72 h of seeding. They were seeded in 96-well plates (5000 cells in each well) and during the last hour, MTS was added to the medium. Each combination of cells was seeded in triplicate wells and analyzed at least three times. The MTS assay was analyzed spectrophotometrically at 490 nm using a 96-well plate reader (Synergy Hi, BioTeck).

### Lentiviral production and infection of NB cells

Lentiviral vectors were produced by transfecting HEK293T cells with the shRNA constructs using Fugene HD transfection reagent (cat#E2311, Promega) following the manufacturer’s instructions. After the removal of cell debris with a 0,22 μm filter, the supernatant containing lentivirus was used to infect NB cells. shRNA mediated knockdown of murine Atg7 was performed using pLKO.1 plasmid (Sigma-Aldrich) carrying Atg7 shRNA#1 and shRNA #2 (TRCN0000305991 and TRCN0000375444) or non-targeting shRNA as control (Sigma-Aldrich, SHC003V). For ATG7 human shRNA (TRCN0000007584), pLKO.1 GFP plasmid (Sigma-Aldrich) is used.

### SiRNAs transfection

RNA interference of ATG7 was performed using Lipofectamine RNAiMAX reagent, in accordance with the manufacturer’s instructions (cat#8054S, ThermoFisher Scientific). Sequence of siRNA oligoribonucleotides againstATG7 was 5'-CAGUGGAUCUAAAUCUCAAACUGAU-3'. After 72 h cells were harvested and subjected to downstream analyses.

### Flow-cytometry

For surface staining, cells were stained with fluorescent labeled antibodies in PBS with 2% FCS for 30 min on ice. Viability was assessed by staining with 7-AAD reagent (cat#559925, BD Bioscience) or DAPI. Samples were analyzed on a Fortessa (BD Bioscience) and LX Cytoflex (Beckman Coulter) flow cytometers and FlowJo software (Treestar, version 10.7.2). All antibodies were purchased from BD Biosciences, Biolegend, Miltenyi Biotec, and the R&D system (listed in Table S1).

### Quantitative real-time PCR

Cells were washed twice with cold PBS and lysed for total RNA extraction using a RNeasy Kit (Qiagen). Extracted RNAs were then used to generate cDNA using MLV reverse transcriptase (Promega). Real-time PCR was carried out in triplicate using SYBR Green PCR Master Mix (ThermoFisher Scientific) on an Applied Biosystem Real-Time. Data were calculated as values relative to GAPDH as control.

### Migration assay

To assess the infiltration capability of GD2.CAR T cells and NT T cells in 3D models of NB cells after autophagy inhibition, 5 × 10^5^ SH-SY5Y-GFP cells were seeded in AggreWell™400 24-well plate (cat#34415, STEMCELL Technologies) and incubated in a humidified atmosphere at 37 °C and 5% CO2 for two days to let hundreds of spheroids form in each well. Then, neo-spheroids were treated with SBI-0206965. After 48 h, GD2.CAR T/NT cells were stained with CellTrace™ Far Red dye for 20 min at 37 °C and added to spheroids at 1:2 E:T. At the final time point, spheroids were recovered, filtered through a 40 μm filter to eliminate single cells, manually disaggregated and T cell infiltration was evaluated using Cytoflex-LX flow cytometer (Beckman Coulter) by counting FarRed positive cells after 8 h of co-culture.

### ELISA assay

Spheroids of SH-SY5Y-GFP cells generated using AggreWell™400 24-well plate (cat#34415, STEMCELL Technologies) and 48 h treated with SBI-0206965 were co-coltured with GD2.CAR T cells (1:2 E:T). After 16 h, supernatants were collected and chemokine CCL5/RANTES was measured using Human CCL5/RANTES DuoSet ELISA kit (cat#DY278, R&D systems), according to the manufacturer’s instructions.

### Subcutaneous tumor model and therapeutic studies

shCtrl and shAtg7 9464D cells (1 × 10^6^) were inoculated sub-cutaneously (s.c.) into the flank of C57BL/6 mice (Charles River). Survival of mice was monitored daily and tumor growth was measured twice weekly using a caliper. Mice were sacrificed after 20 days for IHC, mass-spectrometry, and flow cytometry analyses, and after 5 weeks for both tumor growth and WB analyses.

For chloroquine (CQ) administration, 8 days following tumor implantation when the tumor volume reached 30–50mm^3^, the animals were randomly divided into two groups, and CQ (60 mg/kg) or PBS was injected intraperitoneally (i.p.) every other day for 30 days. Mice were sacrificed after 20 days for IHC and flow cytometry analyses and after 5 weeks for both tumor growth and WB analyses.

All experiments contained 5 to 8 mice per group and were performed at least 2 times, yielding similar results. Animal experiments were conducted at the animal facility Plaisant Castel Romano, Rome, Italy. All animal experiments were following the European Communities Council Directive N. 2010/63/EU, the Italian Ministry of Health guidelines (DL 26/2014), and approved by the Italian Ministry of Health and the local Institutional Animal Care and Use Committee (IACUC) at Istituto Superiore di Sanità (Rome; protocol n° 195/2021-PR).

### Intraperitoneal tumor model and therapeutic studies

To investigate the in vivo antitumor activity of CAR.GD2 T cells, SHSY5Y-FF-Luc.GFP^+^ cells were i.p. injected in NOD-*scid* IL2rg–/– (NSG) female mice (Charles River) (postnatal week 4–5), housed at the animal facility Plaisant Castel Romano, Rome, Italy. The day after engraftment, mice were stratified into two groups, and CQ (60 mg/kg) or PBS were injected i.p. every day for 3 days. For the combination therapy experiment, mice were stratified into the following groups: GD2.CAR T cells + PBS, GD2.CAR T cells + CQ, NT T cells + PBS and NT T cell + CQ. GD2.CAR T cells (NT T-cells were used as control) cultured in vitro for 2 weeks, were resuspended in 100 µL of PBS and infused (10 X 10^6^) once through intravenous (i.v.) injection into the tail vein at day + 4 after tumor inoculation. Mice were then treated with CQ or PBS (used as vehicle control) on alternate days for 12 days. In this model, we considered the mouse to be in disease-free survival (DFS) if the bioluminescence signal was < 1 × 10^9^ p/s/cm^2^/sr and the animal was free of any sufferance sign as previously described [27]. After about 5 weeks, animals were sacrificed, and spleens were collected for T-cell infiltrate analyses by flow cytometry. All animal experiments were following the European Communities Council Directive N. 2010/63/EU, the Italian Ministry of Health guidelines (DL 26/2014), and approved by the Italian Ministry of Health and the local Institutional Animal Care and Use Committee (IACUC) at Istituto Superiore di Sanità (Rome; protocol n° 195/2021-PR).

### Tumor and spleen dissection

Tumors were dissected from mice and a total weight of removed tumor masses was determined. Tumors were cut into small fragments with scissors and then digested in a medium containing 325 KU/ml DNAse I (Sigma) and 1 mg/ml Collagenase III (Worthington Biochemicals) for 30 min at room temperature in agitation followed by 0.1 M EDTA pH 7.2 for additional 5 min. Samples are then filtered through a 70 μm filter, centrifugated, and re-suspended for staining. Spleens were also collected from mice and single cell suspensions were obtained as previously described [28]. Lymphocytes obtained from the spleen were stained for 20 min at 4 °C with a cocktail of fluorochrome-conjugated mAbs (listed in Table S1) and analyzed on Cytoflex-LX (Beckman Coulter) flow cytometer.

### Immunohistochemistry

Tumor masses were fixed in 4% formaldehyde in 0.1 mol/l phosphate buffer (pH 7.2) and paraffin embedded. Sections were cut and stained; sodium citrate (pH 6.0) heat-mediated antigen retrieval was performed and staining was carried out using antibodies directed against CD8 (cat#989415S Cell Signaling). Slides were then mounted using CV Ultra mounting medium (Leica) and assessed blinded to cell identity.

### Mass spectrometry

Dissected tumors were subjected to protein extraction and Trypsin/Lys C digestion using an iST Sample preparation kit (P.O.00001, Preomics) using the standard manufacturer’s protocol. In addition, a further purification step was performed by suspending digested and purified peptides with 50 mM NH4HCO3 (Merck Life Science) 1M urea buffer. Digested peptides derived from 2ug of total extracts were further purified using Zip-Tip (C-18 Resin, ZTC18S096, Millipore), eluted with 80% acetonitrile and 0.1% TFA, dried and suspended in 2.5% acetonitrile, 0.1% TFA, 0.1% formic acid for HPLC separation and high-resolution Q Exactive Plus Orbitrap mass spectrometer (Thermo Scientific) analysis. MaxQuant software (v.1.5.5.1.) was used for protein identification and label-free quantification (LFQ), and Perseus software (v.1.6.12.0) for data analysis, as previously described [29]. The network analysis of DEPs was performed using the String-DB application specifically developed for Cytoscape (v3.8.2) (StringApp). Functional enrichment analysis was achieved by evaluating all identified DEPs for significant GO biological processes and Pathways (KEGG and Reactome) (FDR < 0.05).

### CAR T cell and TCR-T cell experiments

#### Isolation, generation, and transduction of CAR T and TCR-T cells

CAR T cells were produced as described previously [30]. Briefly, peripheral blood mononuclear cells (PBMC) were isolated from healthy donors (University Hospital Würzburg, Würzburg, Germany– Ethical approval number: 250/20) using Ficoll-plaque reagent. Non-tissue culture-treated plates were pre-coated with OKT3 (1 mg/ml) and anti-CD28 (1 mg/ml) mAb. For T cell activation, PBMC were plated in T-cell media consisting of CTS OpTmizer T Cell Expansion medium (ThermoFisher, Waltham, Massachusetts, USA) supplemented with 2.5% human AB serum, 2 mM glutamine, 1% Pen/Strep and cultured in a humidified atmosphere containing 5% CO_2_ at 37 °C. The day after activation, recombinant human IL-7 (500 U/ml) and–IL-15 (50 U/ml) were supplemented. On day two, T cells were transduced as described previously with a third-generation GD2.CAR consists of CD28.4-1BB.zeta-chain (ζ) [27, 31]. Cells were subcultured every 3–4 days and supplemented with fresh T-cell media and cytokines in the concentration indicated above.

TCR-T cells were generated by isolating CD8^+^ T cells from PBMCs using CD8 microbeads (Miltenyi, Bergisch-Gladbach, Germany) and plated on OKT3/CD28-coated non-tissue culture treated plates in complete T-cell media. On day 2 after isolation, cells were treated with IL-7 and IL-15 as indicated above. Three days after isolation, T cells were transduced with a retroviral construct encoding an optimized αβ TCR specific for the HLA-A*02:01 restricted PRAME peptide (425–433, SLLQHLIGL) [32, 33]. Cells were subcultured as described above. Six days after transduction, transduced T-cells were magnetically sorted for PRAME-TCR by labeling with APC-conjugated PRAME-Dextramer (Immudex, Kopenhagen, Denmark) and APC-Microbeads (Miltenyi).

#### Immunophenotype and flow cytometry

Characterization of CAR, TCR, and NT control T cells was performed by flow cytometry. 2–3 × 10^5^ cells were washed twice with FACS buffer (1x PBS, 0.5% BSA, and 2 mM EDTA). For staining of surface markers, cells were incubated for 20 min at 4 °C with the following mAb: CD3-vioblue (Miltenyi Biotec), CD8-FITC (Miltenyi Biotec), 1A7 clone antibody [34] with subsequent secondary Ab anti-mouse PE. For the TCR staining, cells were incubated with APC-conjugated PRAME-specific Dextramer (Immudex) according to the manufacturer’s protocol. For each condition, a minimum of 30,000 events were acquired with FACS Canto II (BD Bioscience) and analyzed using Flowlogic software (Inivai Technologies).

#### Flow cytometry-based cytotoxicity assay

Tumor cells were transduced with a retroviral vector encoding for eGFP as described previously [35]. For co-culture experiments, GD2.CAR-, PRAME.TCR- and control-T cells were cultured with tumor cell lines, SH-SY5Y (NB), THP-1 or HL-60 in RPMI medium supplemented with 10% FBS, 2 mM glutamine and 1% Pen/Strep in an E:T ratio of 2:1. After 4 days, co-cultures were collected and stained for CD3 expression. 7-AAD reagent (Miltenyi) was added and a minimum of 30,000 live cells were acquired with FACS Canto II (BD).

#### MACSima^™^ imaging cycling staining

Tissue sections were obtained from paraformaldehyde-fixed murine spleens embedded in paraffin. The tissue sections were then placed in Xylol at room temperature for 20 min. The deparaffinized tissue was rehydrated through a graded ethanol series (100%, 95%, 80%, 70%, 50%) and water for two minutes each. Tissue sections were preheated at 60 °C for 1 h and antigen retrieval was performed at 98 °C with Trizma-EDTA-Sodium Citrate pH9 buffer for 20 min. MACSwell imaging frames (Miltenyi Biotec) were mounted on the slide and covered with MACSima running buffer (Miltenyi Biotec). Tissue sections were stained with a DAPI solution. Subsequently, sections were iteratively stained using a fully automated MACSima Imaging System (Miltenyi Biotec) with a combination of FITC-, PE, and APC-conjugated Abs. Fluorochromes were photobleached or released using REAlease technology (Miltenyi) after image acquisition at the end of each staining cycle. The following antibodies (all from Miltenyi Biotec) were used: Ki67, CD3, CD4, CD8, CD44, CD45RA, CD45RO, CD57, CD183, CD196, LAG3 (CD223), PD-1 (CD279), TIM3 (CD366), FoxP3, HLA-DR. Acquired images were processed and analyzed by MACSiQ^®^ View Imaging Software (Miltenyi Biotec) following current processing work-flow (Miltenyi Biotec). Processed data were segmented to identify individual cell nuclei using DAPI, cytoplasm using Ki-67 and FoxP3, and cellular membranes using all the other markers as previously reported [36].

### Statistical analysis

GraphPad Prism 9.0.2 software was used to calculate the significance between the samples. Two-tailed Student’s t-test was used to compare the means of the two groups. One-way or two-way ANOVA was used to compare the means of three or more groups. Data for mice survival were presented using Kaplan–Meier survival curves and a log-rank test was performed to determine statistical significance between treatment groups. Unless specifically stated, all data are representative of 3 separate experiments. Error bars represent SEM. The value of *p* ≤ 0.05 was considered to be statistically significant.

## Results

### Autophagy impairment affects NB spheroids’ growth

**Fig. 1:**
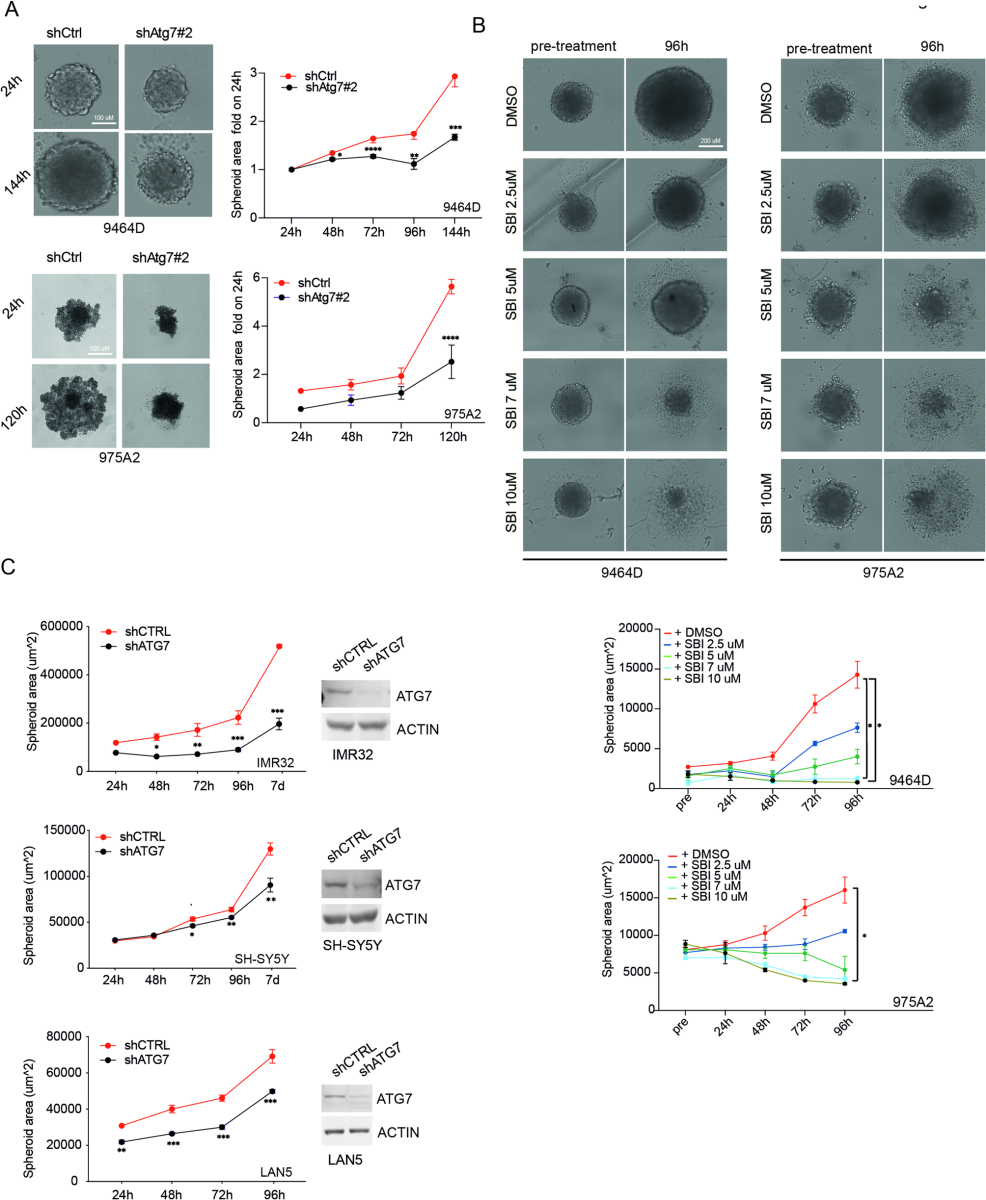
Autophagy inhibition reduces NB growth in 3D spheroids. **(A)** Tumor spheroids were generated from 9464D (top) and 975A2 (bottom) cell lines after *Atg7* downregulation by lentiviral infection (shAtg7#2 or shCtrl). The spheroid area was calculated at the indicated time points. Data show 3D growth as mean ± SEM calculated over 24 h (*p < 0.05, **p < 0.01; ****p < 0.0001; two-way ANOVA *n* = 6). Representative images are shown. **(B)** Tumor spheroids of 9464D and 975A2 cells were generated and treated with the indicated concentration of SBI-0206965. The spheroid area was calculated at the indicated time points. Data show the mean of spheroid area ± SEM calculated at the indicated time points (*p < 0.05, two-way ANOVA, *n* = 3). Representative images are shown. **(C)** Tumor spheroids of IMR32, SH-SY5Y, and LAN-5 cells were generated after *ATG7* downregulation by lentiviral infection (shATG7 or shCTRL). The spheroid area was calculated at the indicated time points. Data show spheroid area as mean ± SEM (*p < 0.05, **p < 0.01; ***p < 0.001; two-way ANOVA *n* = 3). ATG7 down-regulation was assessed by WB. ACTIN was used as a loading control

To address the role of autophagy inhibition on immune responses in NB, we chose two murine NB models, 975A2 and 9464D, derived from TH-MYCN mice as spontaneous NB and immunologically compatible with C57Bl/6 strain [25, 37–39]. We first evaluated the impact of targeting autophagy both genetically (by viral infection with two vectors encoding *Atg7* short hairpin RNA- shAtg7#1 and shAtg7#2) and pharmacologically (by using an inhibitor of ULK1 named SBI-0206965 and chloroquine-CQ) on both 975A2 and 9464D cells grown as 3D spheroids. 3D NB models were reported to better recapitulate the autophagy scenario in NB and therefore allow a better assessment of autophagy-targeting treatment and resistance mechanisms [40]. The efficient knockdown of *Atg7* protein was assessed by Western Blotting analyses and resulted to be more efficient using shAtg7#2 (see Supp. Figure 1A). In addition, SBI-0206965 and CQ treatments resulted in efficient inhibition of both ULK1 kinase activity (assessed by analyzing phosphorylation levels of ATG14) and autophagy flux (by LC3 lipidation) respectively (see Supp. Figure 1B). We investigated the tumor cell viability by live-cell imaging and plate-based microscopy, by measuring the spheroid area after autophagy inhibition. We observed that *Atg7* silencing reduces the size of both 975A2 and 9464D 3D spheroids compared to the control (shCtrl) (Fig. 1A and Supp. Figure 1C). Similar results were obtained using autophagy inhibitors (SBI-0206965 and CQ) at different concentrations (Fig. 1B and Supp. Figure 1D). 9464D cell line results less sensitive to SBI-0206965 compared to the 975A2 cell line as the dose of 5 µM slows cell growth without cytotoxic effects. Comparing drug response between 2D- and 3D-grown 9464D cells, we observed that 3D growth strongly hampered the drug-induced reduction in cell viability compared to 2D growth (Fig. 1B and Supp. Figure 1E). All these results were confirmed using three different human NB cell lines: SH-SY5Y **(**MYCN non-amplified), LAN5 and IMR32 (MYCN amplified). As shown in Fig. 1C and Supp. Figure 1F-G, autophagy inhibition significantly reduces cell growth in 2D, to a lower extent in 3D conditions, in all the analyzed cell lines. It is possible that SBI-0206965 has low penetration through the spheroids, leading to minor growth inhibition even if, in both the 2D and 3D cultures, SH-SY5Y cells are the most sensitive cell line to SBI-0206965. The data indicates that manipulating autophagy effectively inhibits the 3D growth of NB cells, highlighting the significance of this process in this tumoral context.

### Autophagy inhibition increases MHC-I expression in a panel of NB models

**Fig. 2:**
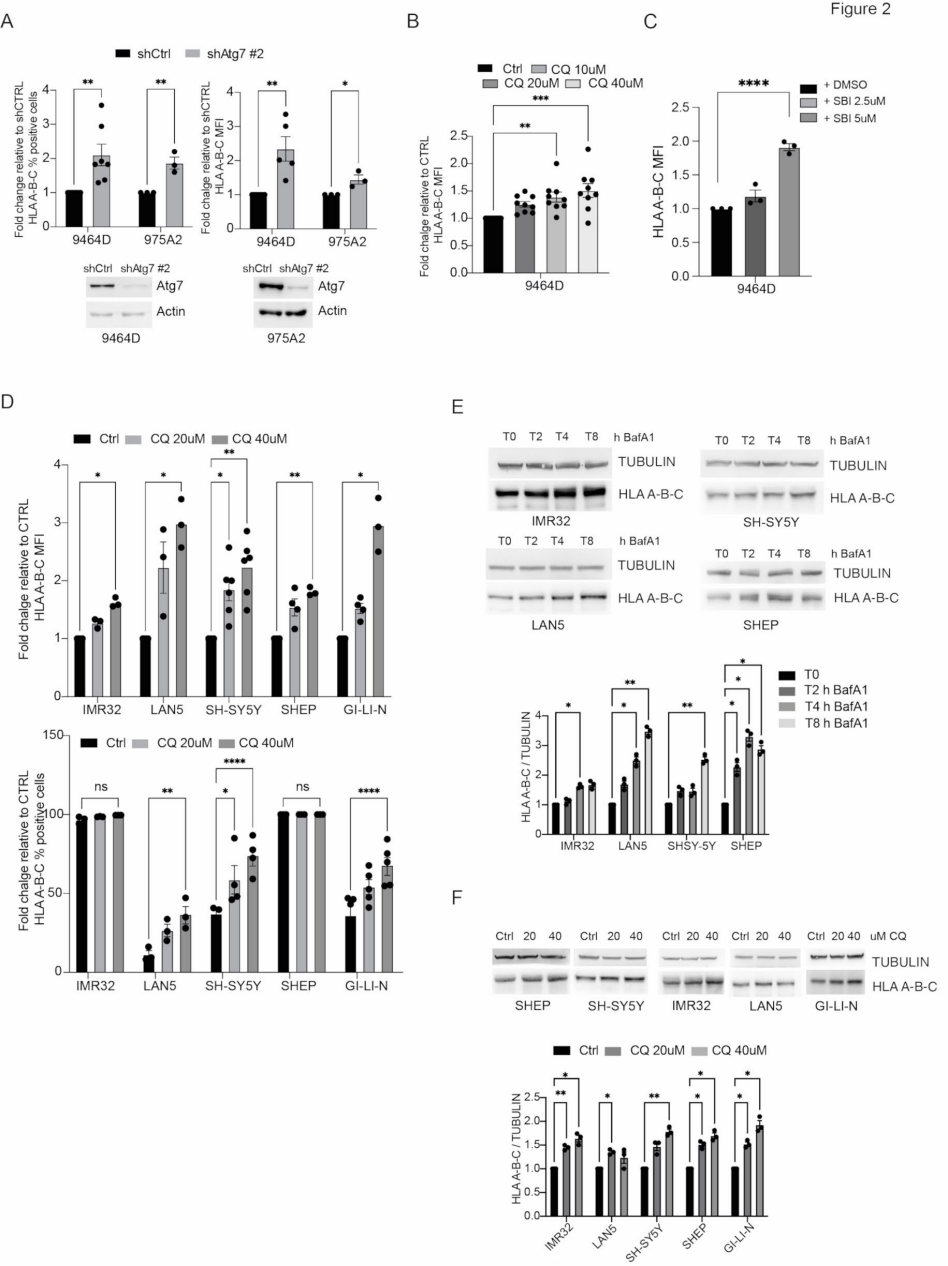
Autophagy inhibition stimulates MHC-I expression in NB. **A)** MHC-I levels were determined by flow cytometry in both 9464D and 975A2 cells after *Atg7* downregulation by lentiviral infection (shAtg7#2 or shCtrl). Data ± SEM are presented as percentage of positive cells (left) and mean fluorescence intensity (MFI) (right) normalized over shCtrl cells (unpaired Student’s t-test). Each dot represents an independent experiment. Representative immunoblotting shows Atg7 protein levels. Actin was used as a loading control; **B-C)** MHC-I expression was determined by flow cytometry after treatment with the indicated doses of CQ (**B**) and SBI-0206965 (**C**) respectively for 48 h in the 9464D cell line. Data ± SEM are presented as mean fluorescence intensity (MFI) normalized over shCtrl cells (one-way ANOVA followed by Tukey post hoc test); **D)** MHC-I expression was determined by flow cytometry in a panel of human NB cell lines after treatment with 20 or 40 µM CQ respectively. Data ± SEM are presented as MFI (top) and percentage of positive cells (bottom) normalized over shCTRL cells (one-way ANOVA); **E-F)** WB analysis of MHC-I protein levels after autophagy inhibition by Bafilomycin A1 (BafA1, 100 nM) or CQ in different human NB cell lines. Representative immunoblotting shows MHC-I expression levels. TUBULIN was used as a loading control. Densitometric analysis of MHC-I expression levels over TUBULIN is shown. Data ± SEM are presented and significance is calculated using two-way ANOVA (*p < 0.05, **p < 0.01; *n* = 3)

Reduced expression of MHC-I molecules is a well-established mechanism contributing to NB resistance to adaptive immunosurveillance and immunotherapy. Similarly, to human high-risk NB, the 9464D murine model expressed low levels of MHC class I and only a few tumor-infiltrating immune cells can be detected inside the tumor mass [37, 39]. Recently it has been reported that MHC-I molecules are selectively targeted for lysosomal degradation by an autophagy-dependent mechanism in pancreatic cancer and this promotes immune evasion [17]. To verify whether autophagy inhibition can modulate the expression of MHC-I molecules in NB, we analyzed, by flow cytometry analysis, the expression levels of MHC-I on the cell surface of murine NB cells (9464D and 975A2) knockdown for *Atg7* or treated with SBI-0206965 and CQ. As shown in Fig. 2A-C blocking of autophagy by both *Atg7* silencing and treatments with SBI-0206965 and CQ significantly increased the surface expression of MHC-I in all the conditions analyzed. We confirmed these results using ULK-101, another selective ULK1 inhibitor able to suppress autophagy and cell growth in different tumoral contexts [41] (Supp Fig. 2A-B). In the same way, SBI-0206965 and CQ treatments increased the surface expression of MHC-I in a panel of human NB cells (Fig. 2D). Finally, lysosomal inhibition with CQ or V-ATPase inhibitor Bafilomycin A1 (BafA1) was able to induce a significant upregulation of total MHC-I protein in all human NB cells analyzed as detected by Western blotting analysis (Fig. 2E). Together, these findings suggest that autophagy inhibition increases the tumor susceptibility to T cell-mediated recognition by up-regulating of MHC class I molecules.

### T cell recruitment in a syngeneic NB model upon autophagy inhibition

**Fig. 3:**
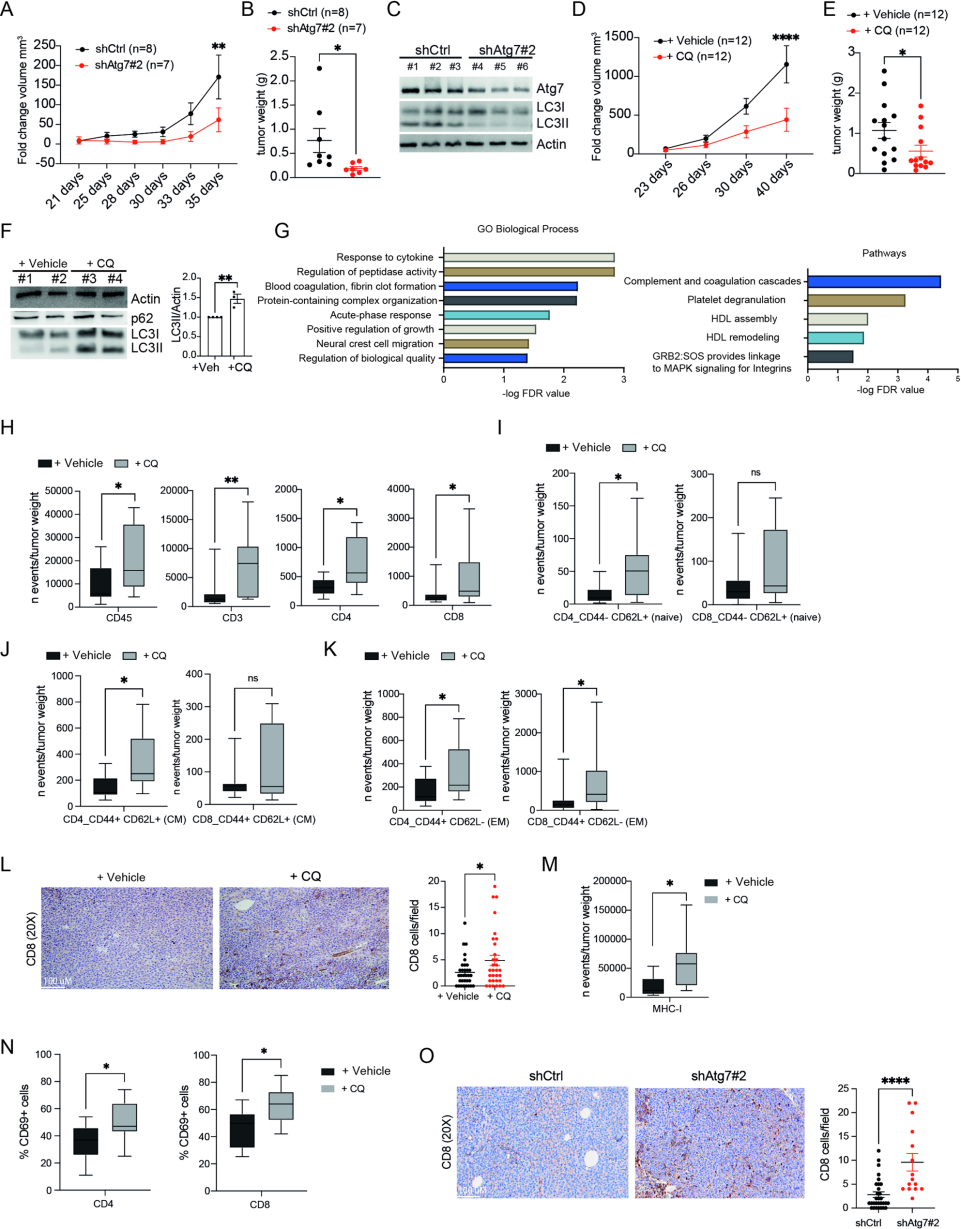
Autophagy inhibition controls tumor growth and increases infiltrating immune cells in a syngeneic mouse model of NB. **(A)** Analysis of tumor volume after subcutaneously (SC) injection of shAtg7 or shCtrl 9464D cells in C57BL/6 mice. Tumor volume was measured by manual calibration at the indicated experimental time points. Statistical analyses by Šídák’s multiple comparisons test. **p < 0.01 **(B)** Analyses of tumor weight. Data ± SEM are presented and analyzed by unpaired Student’s t-test. *p < 0.05. **(C)** Representative immunoblotting of both Atg7 and LC3 in shAtg7 and shCtrl protein extracts from harvested tumors. Actin is used as the internal control. **(D)** Analyses of tumor volume collected from CQ-treated and Vehicle-treated C57BL/6 mice. Tumor volume was measured by manual calibration at the indicated time points. Statistical analyses by Šídák’s multiple comparisons test. ****p < 0.0001). **(E)** Analysis of tumor weight. Data ± SEM are presented and analyzed by unpaired Student’s t‐test. *p < 0.05. **(F)** Representative immunoblotting of p62 and LC3 (LC3 I-II) in protein extracts from harvested tumors. Actin is used as the internal control. LC3 II densitometric analysis in CQ-treated and untreated tumors was shown. Data ± SEM are presented and analyzed by unpaired Student’s t‐test. **p < 0.01. **(G)** GO Biological process and pathway analysis of mass-spectrometry data obtained from protein extracts derived from shAtg7 and shCtrl tumors respectively. **(H)** Flow cytometry analysis of infiltrated immune cells (CD45^+^, CD3^+^, CD4^+^, CD8^+^) in NB tumors treated with CQ or vehicle, and **I-J-K)** CD44^−^/CD62L^+^ (naїve T cells), CD44^+^/CD62L^+^ (central memory T cells, CM), CD44^+^/CD62L^−^ (effector memory (EM) T cells) gated on CD4^+^ or CD8^+^ T cells respectively and normalized on tumor weight. Data are presented as mean ± SEM calculated by Mann Whitney test. *n* > 10. *p < 0.05, **p < 0.01. **L)** Representative IHC staining of CD8^+^ T cells in tumor tissue of CQ-treated and vehicle-treated mice; scale bar: 100 μm. Data are presented as mean ± SEM calculated by unpaired Student’s t‐test. *p < 0.05. **M)** Flow cytometry analysis of MHC-I positive cells normalized on tumor weight in CQ treated mice vs. untreated mice. Data are presented as mean ± SEM calculated by Mann Whitney test. *n* > 10) *p < 0.05. **N)** Flow cytometry analyses of tumor-infiltrating immune cells. Percentage of CD69^+^ cells (activated T cells) gated on CD4^+^ or CD8^+^ T cells collected from 9464D bearing C57BL/6 mice treated with CQ or vehicle. **O)** Representative IHC staining of CD8^+^ T cells in tumoral tissues of shAtg7 cells-bearing mice versus their controls. Scale bar: 100 μm; Data are presented as mean ± SEM calculated by unpaired Student’s t‐test. *p < 0.05

To address the potential impact of autophagy inhibition on NB growth in vivo, we engrafted 9474D shCtrl and shAtg7#2 cells in the left flank of syngeneic C57BL/6 mice. As shown in Fig. 3A-B, *Atg7* silencing resulted in significant inhibition of tumor growth as compared to control mice (shCtrl). To confirm that the data observed were related to the autophagy block, a Western Blotting analysis was carried out. The analysis revealed *Atg7* downregulation and consequently autophagy inhibition (by assessing the conversion of LC3-I to LC3-II) in 9474D shAtg7#2 cells compared to the control cells (shCtrl) (Fig. 3C). As next step, we evaluated the impact of pharmacological targeting of autophagy on tumor growth by systemic treatment of 9474D engrafted mice with CQ (60 mg/kg). As reported in Fig. 3D-E, CQ treatment resulted in significant inhibition of tumor growth and a reduction in tumor weight. Autophagy inhibition was confirmed by Western Blotting analysis (Fig. 3F). Subsequently, we explored whether this autophagy-dependent anti-tumor activity was linked to a modulation of the tumor immune infiltration. Therefore, we performed a mass spectrometry analysis on tumoral samples (shAtg7#2 *versus* shCtrl) isolated ex vivo from tumor-bearing animals. We identified a set of differentially expressed proteins that were significantly modulated (FDR < 0.05) in *Atg7*-depleted tumors (Fig. 3G and Supp. Figure 3A-B) respect to the control. Most of the altered pathways including complement and coagulation cascades (KEGG-based pathway), response to cytokines, and acute-phase response (Biological process-based pathway) are involved in the imbalance of inflammation and immune cells, supporting the crucial role of autophagy in regulating tumor immune landscape in NB.

Based on these findings, we conducted a comprehensive analysis of the different immune effector cell subsets infiltrating 9474D tumors (of 100–150 mm^3^) after CQ treatment using flow cytometry. We showed that the number of live CD45^+^ and CD3^+^ cells was significantly increased in CQ-treated tumors as compared to the control group (+ Vehicle) (Fig. 3H). In the CD3 T cell population, both CD4^+^ and CD8^+^ compartments were significantly increased in terms of the absolute number of cells for grams of tumor. Furthermore, a significant frequency increment of naïve, central memory and effector memory was observed in the CD4^+^ population, while in the CD8^+^ cells, although a trend was found in all the subpopulations, only the effector memory compartment (CD44^+^/CD62L^−^) was significantly augmented (Fig. 3I-K). The heightened infiltration of CD8^+^ T cells into CQ-treated 975A2 tumors was additionally confirmed by IHC analysis on five distinct tumor sections (Fig. 3L). According to the in vitro results, we found a significant increase in MHC-I expression levels on CQ-treated tumor cells (Fig. 3M). Moreover, a significant increase in the expression level of the activation marker CD69 in both CD4⁺and CD8⁺ T cell populations was also detected (Fig. 3N). Additionally, PD1 expression was found to be upregulated within the CD69⁺subset in CD8⁺T cell populations following CQ treatment, supporting their activated status (Supp. Figure 3C). We did not observe enrichment of CD69⁻PD1⁺cells, suggesting no clear signs of T cell exhaustion under these conditions. We further validated our findings using *Atg7#2* silenced tumor cells. As shown in Fig. 3O, a significant increase of infiltrated CD8^+^ T cells was detectable within shAtg7 975A2 tumors by IHC on three distinct tumor sections. Flow cytometry analysis revealed an increasing trend for T cells. A more detailed analysis of the CD3 compartment showed enrichment of effector memory T cells, though this increase reached statistical significance only within the CD4^+^ T cell subset (Supp. Figure 3D-E). Overall, these data indicate that autophagy impairment increases the recruitment of cytotoxic CD8^+^ T cells inside the tumoral masses.

### Autophagy suppression sensitizes NB cells to T cell-mediated cytotoxicity

**Fig. 4:**
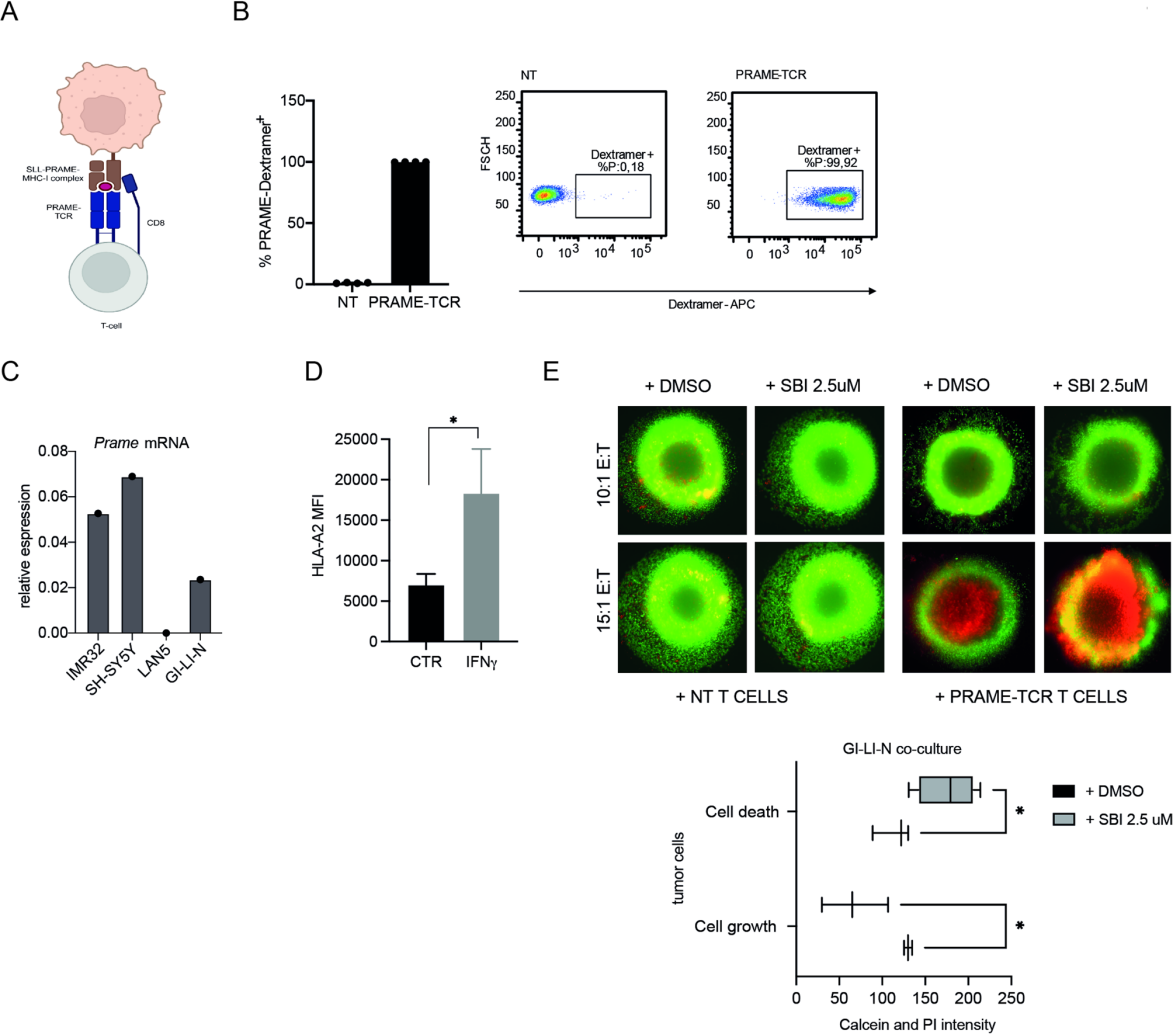
Autophagy inhibition sensitizes NB cells to T cell-mediated cytotoxicity. **A**) Schematic representation of CD8^+^ T cells transduced with a transgenic PRAME-TCR specific for the SLL-PRAME_(425−433)_ peptide. **B**) PRAME-TCR expression levels on NT and TCR-sorted cells. Cells were surface stained with an APC-conjugated PRAME-Dextramer (*n* = 4). Representative dot plot of NT control and PRAME-TCR transduced T cells. **C**) *PRAME* gene expression determined by qPCR of NB cell lines relative to GAPDH. **D**) HLA-A2 MFI were measured by flow cytometry in GI-L-IN cells in the presence or absence of IFNγ. **E**) Representative images and average (AVG) of calcein (Green) and PI (Red) intensity of 24 h SBI-0206965 (SBI) treated GI-LI-N spheroids co-cultured with NT and PRAME-TCR T cells at two different E:T z ratios and for the indicated time points (*n* = 3 different donors). Data ± SEM are presented and analyzed by unpaired Student’s t-test. *p < 0.05

Next, we explored whether the upregulation of MHC-I induced by autophagy inhibition was able to enhance the activation and cytotoxicity of primary human T lymphocytes. Since NB has been proved to overexpress the preferentially expressed antigen of melanoma (PRAME) [42], HLA-A2 restricted PRAME-SLL specific αβTCR-T cells were generated. PRAME, indeed, was initially identified as a gene encoding antigens presented by HLA-A*24, capable of activating tumor-specific cytotoxic T lymphocytes (CTLs) derived from melanoma patients [43]. After retrovirus transduction, CD8^+^ T cells were sorted and stained with the SLL dextramer (Fig. 4A-B and Supp. Figure 4A) and tested as effector against THP-1 (PRAME^+^ and HLA-A2^+^) and HL-60 (PRAME^+^ and HLA-A2^−^) target cells in an E:T ratio of 1:2 (Supp. Figure 4B).

Next, to investigate the effect against NB cells, GI-LI-N cell line was selected based on the presence of both PRAME and the HLA-A2 haplotype (Fig. 4C-D). GI-LI-N cells express low levels of HLA-A2 molecules which can be upregulated by IFNγ treatment (Fig. 4D). Therefore, we then assessed the cytotoxic activity of PRAME-SLL and mock T cells against GI-LI-N tumor spheroids. After spheroid formation (48 h), cells were exposed to SBI-0206965 (2.5 μM) for 24 h and then co-cultured with PRAME-SLL or mock T cells respectively. The cytotoxic potency of the effector cells was tested at E:T ratios of 15:1 and 10:1 and monitored after 5 days by staining the spheroids with calcein (green, vitality marker) or PI (red-dead marker). As shown in Fig. 4E, pre-treatment with SBI-0206965 resulted in an increased cytotoxic capacity of T cells, as assessed by the decrease in calcein and the increase in PI staining, respectively. In selected experiments, the co-culture experiments were performed in the presence of SBI-0206965. Again, we observed anti-tumor activity of PRAME-specific T cells, indicating that SBI-0206965 did not affect the cytotoxic activity of T cells.

### Targeting autophagy enhances CAR T cell-mediated killing of NB cells

Autophagy is considered a critical cell survival mechanism driving NB tumorigenesis and leading to its resistance to chemotherapy [44, 45] Both MYCN non-amplified and MYCN amplified NB cell line are known to express GD2. It has been reported that targeting autophagy can sensitize NB to anti-GD2 immunotherapy treatment [46]. Based on these observations, we investigated the effects of blocking autophagy in NB, both genetically via targeting the *ATG7* gene and pharmacologically via treatment with SBI-0206965 and CQ, on GD2.CAR T cell-mediated therapy. We used SH-SY5Y and CHLA255 cell lines that are known to express the GD2 antigen. T cells were transduced with third-generation GD2.CAR [15, 27]. The transduction efficiency was assessed by flow cytometry while their anti-tumor capacity was tested by long-term co-culture experiments (Supp. Figure 5A-C).

**Fig. 5:**
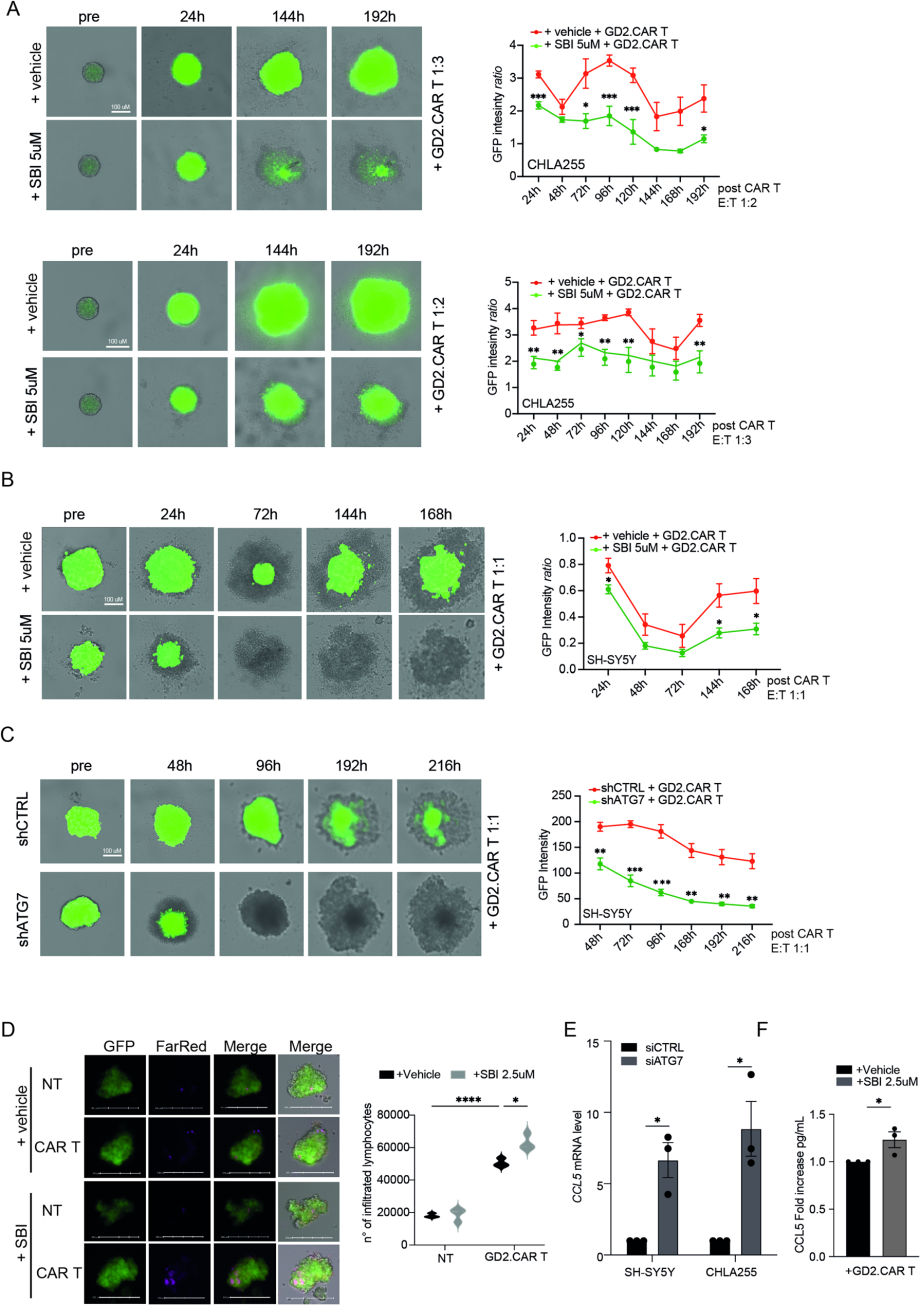
Autophagy inhibition enhances the susceptibility of NB spheroids to CAR T cell-based immunotherapy. **A-B)** Long-term co-culture killing assays. Representative images and average (AVG) GFP intensity of 24 h SBI-0206965 (SBI)-pretreated CHLA-255-GFP^+^ spheroids co-cultured with GD2.CAR T cells at two different E:T ratios and for the indicated time points. Data (mean of GFP fluorescence intensity (au) ± SEM) are presented as the ratio of each time point compared to pre-treatment. Significance is calculated by two-way ANOVA (*n* > 3 experiments). *p < 0.05, **p < 0.01, ***p < 0.001. **(C)** Long-term co-culture killing assays. Representative images and average (AVG) GFP intensity of 24 h SBI-0206965 pretreated SH-SY5Y-GFP^+^ spheroids co-cultured with GD2.CAR T cells at 1:1 E:T ratio and for the indicated time points. Data (mean of GFP fluorescence intensity (au) ± SEM) are presented as the ratio of each time point with respect to pre-treatment. Significance is calculated by two-way ANOVA (*n* > 3 experiments). *p < 0.05. **C)** Long-term co-culture killing assays. Representative images and AVG GFP intensity of shATG7 vs. shCTRL SH-SY5Y-GFP^+^ spheroids co-cultured with GD2.CAR T cells at E:T ratio of 1:1 for the reported time points. Data (mean of GFP fluorescence intensity (au) ± SEM) are shown. Significance is calculated by two-way Anova (*n* > 3 experiments). **p < 0.01, ***p < 0.001. **D)** Representative images of GD2.CAR T cell infiltration (labeled with FarRed) in the 3D model of SH-SY5Y-GFP^+^ is shown. GD2.CAR T cells or NT T cells (1:2 E:T) were analyzed by flow cytometry in SH-SY5Y-GFP^+^ spheroids pre-treated with 2.5µM SBI-0206965 for 48 h. Data are presented as mean ± SEM of three independent experiments. Statistical analyses by two-way ANOVA. **p < 0.01, ****p < 0.001. **E)** qPCR of *CCL5* mRNA in CHLA-255 and SH-SY5Y cell lines after 72 h silencing using siRNA for ATG7 (siATG7) or unrelated control (siCTRL); **F)** ELISA quantification of CCL5 in the supernatant of SHSY-5Y-GFP^+^ spheroids pre-treated with SBI-0206965 (2.5 µM) and co-cultured with GD2.CAR T at 1:2 E:T ratio for 16 h. Data are presented as mean ± SEM of three independent experiments. Statistical analyses by unpaired Student’s t-test. *p < 0.05, **p < 0.01

To assess the cytotoxic effects of GD2.CAR T and mock T cells against SH-SY5Y-GFP^+^ and CHLA255-GFP^+^ tumor spheroids, image cytometry was employed. Before this, spheroids monoculture’s sensitivity to SBI-0206965 and CQ was tested to determinate the optimum concentration that would produce a mild chemotoxic effect, in order to maintain acceptable spheroid viability and aggregation, crucial conditions to assess any synergistic effects produced by CAR T cells in all the conditions (Supp. Figure 5D). After formation, spheroids were exposed to SBI-0206965 for 24 h, after which GD2.CAR T or mock T cells were added. The cytotoxic potency of the effector cells was tested at 1:3, 1:2, and 1:1 E:T ratios and monitored by measuring the average GFP MFI of the spheroids from 0 to 192 h in 24-hour intervals. As shown in Supplementary Fig. 5E-F, untreated spheroids, as well as those treated with mock T cells, showed no significant changes in tumor cell viability or spheroid size over time. In contrast, GFP fluorescence in spheroids treated with GD2.CAR T cells at a 1:1 E:T ratio further decreased even in the absence of SBI-0206965, confirming that GD2.CAR T cells effectively target the GD2 antigen on both SH-SY5Y-GFP^+^ and CHLA255-GFP^+^ tumor spheroids. However, at lower E:T ratios of 1:3 and 1:2—representing the physiological ratios observed in patients undergoing GD2.CAR T-cell therapy—spheroid size was significantly reduced only in the presence of SBI-0206965, thus indicating the potential role played by autophagy inhibition in combination with CAR T cell-mediated therapy (Fig. 5A, B and Supp. Figure 5G). Between the cell lines used, SH-SY5Y are the most sensitive to GD2.CAR T cells. Next, we validated the results exposing both SH-SY5Y-GFP^+^ and CHLA255-GFP^+^ spheroids to CQ for 24 h and adding GD2.CAR T at 1:3 E:T ratio. As shown in Supplementary Fig. 5H-I, spheroid size was significantly reduced in the presence of CQ. Finally, we confirmed the results after genetic inhibition of autophagy by using SH-SY5Y-GFP^+^ shCTRL and shATG7 cells respectively. As shown in Fig. 5C and Supp. Figure 5J, in all the E:T ratios analyzed, there is a significant reduction of GFP fluorescence in shATG7 cells post-addition of GD2.CAR T cells compared to the control (shCTRL).

Since cell invasion in a complex 3D TME is critical for the engagement of cytotoxic T cells and for the achievement of their efficient anti-tumor activity, we decided to study the effects of autophagy inhibition on NB spheroids in regulating GD2.CAR T cells invasion capability. SH-SY5Y-GFP^+^ spheroids were cultured in a microwell array and treated with SBI-0206965 for 48 h. Then, GD2.CAR T or mock T cells, labeled with a far-red dye, were added an E:T ratio 1:2 for 8 h, to allow T cell infiltration without tumor spheroid destruction. Isolated tumor spheroids were then disrupted by enzymatic digestion and analyzed by flow cytometry. As shown in Fig. 5D, we found that GD2.CAR T cell infiltration was enhanced within NB spheroids following SBI-0206965 treatment, suggesting that targeting autophagy facilitates GD2.CAR T cell infiltration in NB cells.

Some mechanistic links between autophagy and the secretion of proteins, including cytokines, have been already described [47]. In particular, both CCL5/Rantes and CXCL10/IP10 mRNA, and their corresponding secreted proteins, were significantly up-regulated in autophagy-deficient melanoma cells. Further, CCL5 blocking significantly rescued the inhibition of tumor growth and was associated with a significant decrease in the infiltration of both NK and CD8 T cells [48]. Interestingly, the use of oncolytic adenoviral vectors expressing CCL5 and GD2.CAR T cells has been reported to robustly control NB progression and improve CAR T cell efficacy and migration in mice [49]. We thus evaluated the expression of *CCL5* after genetic autophagy downregulation in NB cells (using siRNAs against ATG7#2) observing an increase in *CCL5* mRNA levels (Fig. 5E). To analyze whether pharmacological inhibition of autophagy (by SBI-0206965) could increase CCL5 production and secretion, we quantified CCL5 protein by ELISA in the supernatant of SH-SY5Y-GFP^+^ spheroids pre-treated (or not) with SBI-0206965 and co-cultured which GD2.CAR T for 16 h. As shown in Fig. 5F, CCL5 was significantly up-regulated in SBI-0206965-treated SH-SY5Y-GFP^+^ spheroids compared to control ones, suggesting that autophagy inhibition induces the secretion of pro-inflammatory chemokines as CCL5 that could promote the recruitment of CAR T cells into the TME.

### Functional targeting of autophagy sensitizes NB to CAR T-cell therapy in vivo

**Fig. 6:**
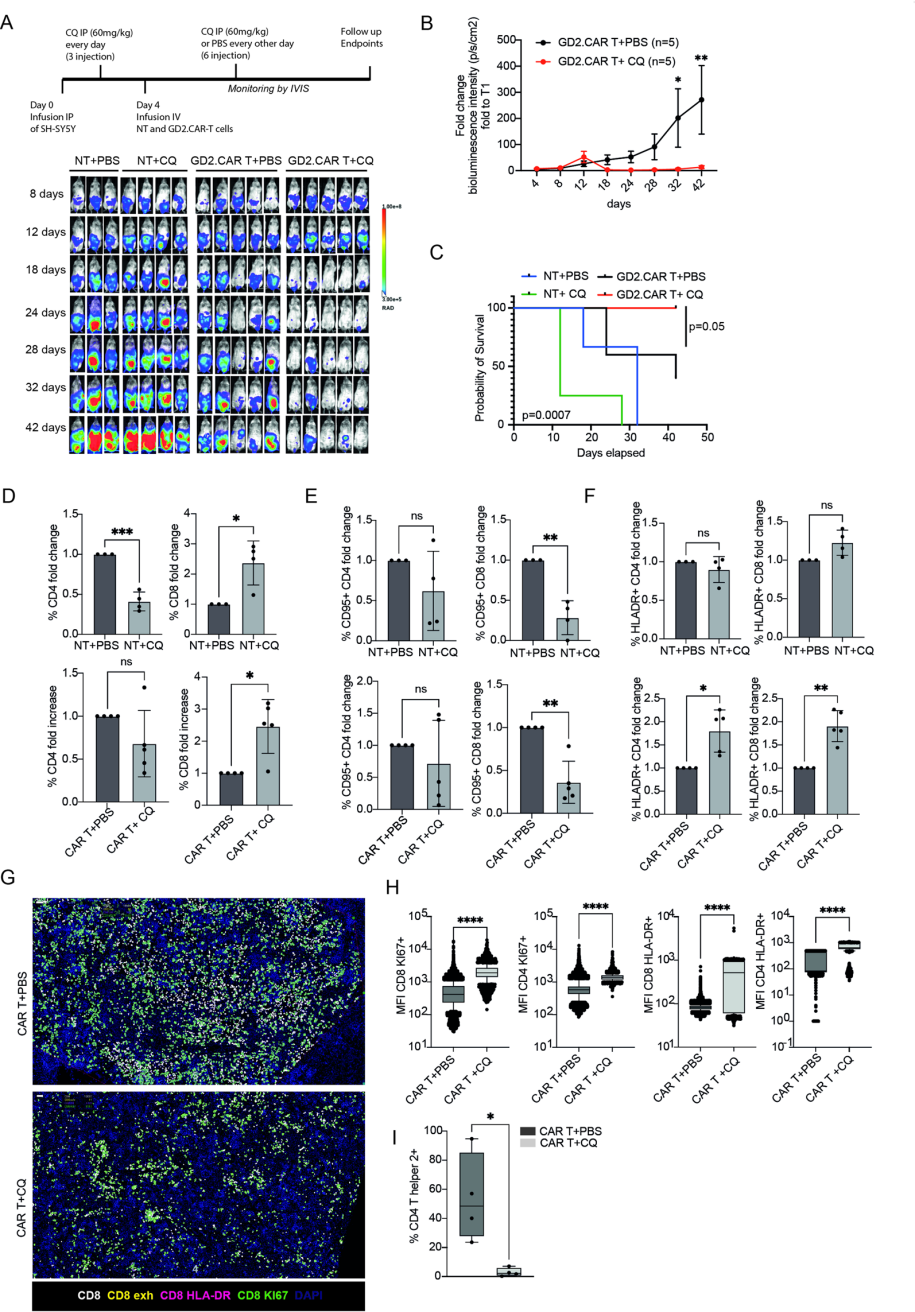
In vivo vulnerability of NB to CAR T-cell therapy after autophagy inhibition. **(A)** Draft and representative bioluminescent imaging of in vivo administration of GD2.CAR T cells and NT T cells in NOD-SCID mice intraperitoneally (IP) injected with SHSY5Y-FF-Luc.GFP^+^ cells and treated with PBS or CQ. Tumor growth was monitored at different time points for in vivo bioluminescence acquisition. **(B)** Tumor growth analyses and **(C)** survival analysis in mice receiving GD2.CAR T or NT cells alone or combined with PBS or CQ. Statistical analyses by two-way ANOVA followed by Šídák’s multiple comparisons test. **(D)** Percentage of CD4^+^, CD8^+^, CD95^+^, and HLA-DR^+^ cells isolated from spleens of NT + PBS versus NT + CQ, or in GD2.CAR T + PBS versus GD2.CAR T + CQ receiving mice respectively. Spleens were collected at the final time point and flow cytometry analyses were performed. Statistical analysis by unpaired Student’s t-test. *p < 0.05, **p < 0.01. **G**) Representative images using MICS technology of spleen tissue sections from GD2.CAR T + PBS and GD2.CAR T + CQ treated mice with indicated stain markers. Scale bars: 40 μm. **H**) Mean fluorescent intensities (MFI)/cell of Ki67 and HLA-DR in both CD4^+^ and CD8^+^ T cell subpopulation. Statistical analysis by unpaired Student’s t‐test. ****p < 0.001. **I)** Frequency of positive T Helper2 cells in the CD4^+^ population. Statistical analysis by unpaired Student’s t‐test. *p < 0.05

We further investigated the effect of autophagy signaling on the anti-tumor activity of GD2.CAR T cells in vivo. NOD-SCID mice were engrafted intraperitoneally with GD2^+^ SH-SY5Y-FF-Luc.GFP cells mixed with Matrigel, so allowing the formation of complex and structured tumors. Next, mice were randomized into four groups: those engrafted with non-transduce T cells plus PBS (NT + PBS) or CQ (NT + CQ, 60 mg/kg i.p.) and two groups engrafted with GD2.CAR T cells plus PBS (CAR T + PBS, i.v. after 4 days) or CQ (CAR T + CQ, i.v. after 4 days) (Fig. 6A). As demonstrated by the bioluminescence data (Fig. 6A-B and Supp. Figure 6A), independently of CQ treatment, mice receiving NT cells failed to control tumor growth. Conversely, GD2.CAR T cell-treated mice demonstrated a significant anti-tumor response. Notably, pretreatment with CQ was capable to induce a more pronounced and significant improvement in the anti-tumor response as evidenced by data in Fig. 6B. Interestingly, based on the > 1 × 10^9^ total flux cutoff (photons per second), we observed a significant improvement in the overall survival of mice treated with CQ and GD2.CAR T cells (CAR T + CQ), compared to the NT-treated groups (NT + PBS and NT + CQ) (*p* = 0.007), and to those treated with CAR T + PBS (*p* = 0.05) (Fig. 6C). Among the groups treated with GD2.CAR T cells, tumors were detectable in one out of 5 (20%) of the CQ-treated mice, and 3 out of 5 (60%) of the vehicle-treated mice. Moreover, flow cytometry confirmed a marked decrease in tumor cells within the spleens of GD2.CAR T cell-treated mice compared to NT cell-treated controls (Supp. Figure 6B-C). Remarkably, the flow cytometry analysis of T cells revealed an increased presence of total CD8^+^ T cells in CQ-treated tumors, independently of CAR expression (Fig. 6D). Additionally, we observed a significant decrease in CD8^+^ CD95^+^ T cells in CQ-treated mice while a significant increase in CD8^+^ HLA-DR^+^ T cells was detected exclusively in mice treated with GD2.CAR T cells, compared to those receiving NT cells (Fig. 6E-F). A similar analysis performed on CAR^+^ cells showed an increase of GD2.CAR T cells only in CQ-treated mice, with a significant enrichment of CD8^+^ population (Supp. Figure 6D-E). Additionally, the HLA-DR activation marker was enhanced for both CD4^+^ GD2.CAR T cells and CD8^+^ GD2.CAR T cells after CQ treatment (Supp. Figure 5F).

In order to consolidate and further validate these results, on the same murine spleens a spatial proteomic was carried out. Several human T cell lineages as well as activation and exhaustion markers were analyzed (Fig. 6G). As reported in the Suppl. Figure 6G-H and Fig. 6H, no difference was observed in terms of absolute frequency of activated and proliferating T cells. However, mice treated with CQ and GD2.CAR T cells display, in both CD4^+^ and CD8^+^ T cells, a significant increase in the number of HLA-DR and Ki67 molecules (Fig. 6H). It is important to note that this increased expression of activation and proliferation molecules, in both T subpopulations, did not lead to a modification of other activation molecules such as PD1, Lag3, and Tim3 and, above all, did not induce exhaustion (defined as PD1^+^Lag3^+^TIM3^+^) (Suppl. Figure 6I-J). Furthermore, treatment with CQ and GD2.CAR T cells showed a significant reduction in the Th2 population defined as CD4^+^CD196^−^CD183^−^ (Fig. 6I). Together, these findings suggest that the combination of GD2.CAR T cells and CQ enhances anti-tumor activity by improving T cell persistence and function, even 40 days post-infusion. Additionally, CQ treatment reduces Th2^+^ CD4^+^ T cells, which support tumor eradication, while positively shaping the TME.

## Discussion

High-risk NB are classified as immunologically “cold” tumors due to the low numbers of infiltrating T-cells, low MHC-I expression, and low mutational burden leading. This is due to an immunosuppressive TME, a key factor limiting the effectiveness of immunotherapy. Many efforts are underway to transform “cold” tumors into “hot” ones, by enhancing T-cell infiltration and employing adaptive therapies, such as CAR T cells [18, 50, 51]. Consequently, understanding the critical factors that influence T-cell infiltration into tumors is essential for developing effective therapies targeting “cold” tumors. In this work, our results suggest that autophagy is a crucial regulator of immunogenicity in NB. In particular, we found that genetic and pharmacological inhibition of autophagy can increase cell surface MHC-I expression and induce the recruitment of activated T cells into the TME. This is in line with a gene expression correlation study [52] that firstly compared autophagy-related genes (ATGs) between INSS stage 4 and other stages of NB patients revealing a significant association between ATGs risk signature and NB immune microenvironment. Given the critical roles of several autophagy mediators in the regulation of chemoresistance [53–56], angiogenesis [57], metabolism [58] and metastasis [59] in NB, our findings on immune evasion contribute to the expanding body of evidence for both cell-autonomous and non-cell-autonomous roles of the autophagy in NB pathogenesis. In the context of the immune response, several studies reported that targeting autophagy could sensitize tumors to immune checkpoint inhibitors combined treatments, or to adoptive cell therapy, in adult cancers. Evidence of autophagy modulation in pediatric solid tumors is limited and, as in adults, its effects could be tumor-specific; however, its manipulation remains an exciting candidate strategy for combined treatments. After confirming that autophagy supports NB cell growth and viability in both 2D and 3D conditions, this study demonstrates that genetic and pharmacological inhibition of autophagy is able to increase MHC-I expression on NB cells. MHC-I molecules are crucial for the recognition by the immune system of cells altered by virus or neoplastic transformations, and as part of their physiological regulations they are continuously removed from the cell surface and degraded or recycled intracellularly. Recently, it was found that the immunity-related GTPase family Q protein (IRGQ) acts in the quality control of MHC class I molecules by mediating their autophagy-dependent lysosomal degradation through binding to GABARAPL2 and LC3B [21]. Our findings provide additional support for the importance of autophagy-dependent degradation of MHC-I in contributing to T cell-mediated tumor recognition and cytotoxicity, suggesting a crucial role of autophagy as a negative regulator of the adaptive immune response in NB.

In the last years, transcriptional and epigenetic analyses of NB tumors have revealed the existence of two distinct cellular identities, named adrenergic (ADRN) and mesenchymal (MES), which have been linked to chemo- and immune-resistance [60, 61]. Intriguingly, conversion from the adrenergic to the mesenchymal cell state has been reported to be sufficient to induce the expression of tumor cell-intrinsic immune genes (such as genes involved in antigen processing and presentation), to increase cell-surface MHC and ligands for the NKG2D receptor [62]. Our data show that autophagy inhibition can upregulate MHC-I expression in both MES (SHEP) and ADRN (SH-SY5Y, LAN5, IMR32) cell lines. Since many studies have demonstrated the interplay between anti-tumor immune responses and autophagy, via several mechanisms [63], we could speculate that autophagy levels differ in ADRN versus MES cells and that autophagy manipulation could have an impact on the conversion from one to the other cell state. But this should be experimentally confirmed.

CAR T-cell therapy has revolutionized cancer treatment, showing remarkable success, particularly in addressing hematological malignancies. However, the effectiveness of this therapy in solid tumors has been hindered by challenges such as tumor antigen heterogeneity, limited CAR T cell trafficking and infiltration, and the presence of an immunosuppressive TME. To date, numerous studies have demonstrated that combining CAR T-cell therapy with other therapeutic approaches can significantly enhance its efficacy while mitigating associated toxicities [64]. In this study, we provide the first evidence that autophagy inhibition can enhance the efficacy of GD2.CAR T cell-based therapy in a solid tumor such as NB. Using co-cultures with NB spheroids, we demonstrate that the addition of an autophagy inhibitor (SBI-0206965 or CQ) significantly boosts the cytotoxic activity of GD2.CAR T cells. Consistently, *ATG7* gene knockout yielded similar results, further validating the protective role of autophagy in NB. Furthermore, flow cytometry analysis confirmed that treatment with SBI-0206965 enhanced GD2.CAR T cells infiltration into NB spheroids. Notably, in vivo experiments revealed that pharmacological inhibition of autophagy by CQ attenuated resistance to GD2.CAR T cells in mice engrafted with NB cells.

Since CAR T cell recognition of tumor cells is independent of MHC molecules, the precise mechanism by which autophagy modulates NB resistance to CAR T-mediated killing remains to be elucidated. Furthermore, following the recent approval of Afamitresgene autoleucel, the first TCR T cell product [65], we extended our findings to an HLA-dependent context. Specifically, we demonstrated that PRAME-TCR redirected T lymphocytes were able to elicit an effective anti-tumor response in 3D spheroids of NB cells characterized by low HLA-Class I expression only when autophagy was inhibited.

From a clinical point of view, although SBI-0206965 is a well-characterized tool for investigating early-stage autophagy inhibition, its in vivo applicability is limited, due to poor absorption following oral administration and low systemic exposure after intraperitoneal dosing in mice [41]. ULK1 has emerged as a promising target for autophagy inhibition because of its central role in initiating the pathway, its druggable nature, and its apparent specificity for regulating autophagy. In contrast, chloroquine is one of the few autophagy inhibitors with regulatory approval and established tolerability in humans, although it lacks strong specificity for the autophagy pathway. While the therapeutic targeting of ULK1/2 is emerging as a promising strategy in cancer, most available inhibitors remain at the preclinical research stage. Notably, DCC-3116 is the only ULK1/2 inhibitor currently undergoing clinical evaluation (NCT04892017). Although its reported limited brain penetration may pose challenges for central nervous system tumors, it would be of great interest to investigate its in vivo efficacy in NB or other relevant models in future studies.

Our findings highlight the critical importance of autophagy as a cancer cell-intrinsic mechanism that significantly limits the CAR T-cell therapy in NB, suggesting that targeting autophagy may offer a promising avenue for improving treatment outcomes for NB patients.

## Conclusions

Our study highlights the crucial role of autophagy as a negative regulator of the immune response in NB, limiting both T cell infiltration and CAR T-cell efficacy. By demonstrating that autophagy inhibition enhances MHC-I expression and facilitates immune recognition, we provide novel insights into overcoming the immunosuppressive TME. Importantly, we show that targeting autophagy significantly improves the cytotoxic activity of GD2.CAR T cells in 3D models and in vivo. These findings suggest that combining CAR T-cell therapy with autophagy inhibitors could be a promising strategy to enhance treatment efficacy in NB. Further studies are warranted to elucidate the precise mechanisms linking autophagy to immune evasion and to optimize combinatorial therapeutic approaches.

## Electronic supplementary material

Below is the link to the electronic supplementary material.

Supplementary Material 1

Supplementary Material 2

Supplementary Material 3

## Data Availability

No datasets were generated or analysed during the current study.

## Abbreviations

BafA1: Bafilomycin A1
CAR T: Chimeric Antigen Receptor T cells
CD: Cluster of differentiation
CQ: Chloroquine
CTL: Cytotoxic T lymphocytes
E: T: Effector:Target ratios
GFP: Green fluorescent protein
HLA: Human leukocyte antigen
IFNγ: Interferon-γ
MFI: Mean fluorescence intensity
MHC-I: Major histocompatibility complex class I
NB: Neuroblastoma
NK: Natural killer cells
NT cells (or Mock cells): Non-transduced T cells
SBI-0206965: ULK1 inhibitor
TCR: T cell receptor
TME: Tumor microenvironment
