# Correction: Inhibition of autophagy enhances the antitumor efficacy of T/CAR T cell against neuroblastoma

**DOI:** 10.1186/s13046-025-03492-7

**Published:** 2025-08-04

**Authors:** Francesca De Mitri, Manuela Giansanti, Ombretta Melaiu, Dorothee Haas, Stefan Ebert, Nicola Tumino, Elisabetta Vulpis, Francesca Gatto, Beatrice Martuscelli, Manuela Antonioli, Elisabetta Sangiuliano, Simona Caruso, Marco Scarsella, Cristiano De Stefanis, Veronica Marabitti, Silvia Campello, Doriana Fruci, Paola Vacca, Ignazio Caruana, Francesca Nazio

**Affiliations:** 1 Department of Biology, University of Rome Tor Vergata, Rome, Italy; 2 Innate Lymphoid Cells Unit, Bambino Gesù Children’s Hospital IRCCS, Rome, Italy; 3 Department of Pediatric Hematology and Oncology of Cell and Gene Therapy, Bambino Gesù Children’s Hospital IRCCS, Rome, Italy; 4 Department of Clinical Sciences and Translational Medicine, University of Rome “Tor Vergata”, Rome, Italy; 5 Department of Pediatrics - Hematology, Oncology and Stem Cell Transplantation Unit, University Hospital Würzburg, Würzburg, Germany; 6 Department of Laboratory Medicine, Division of Pathology, Karolinska Institute, Huddinge, Sweden; 7 National Institute for Infectious Diseases, “Lazzaro Spallanzani” - IRCCS, Rome, Italy; 8 Research Laboratories, Bambino Gesù Children’s Hospital IRCCS, Rome, Italy

**Correction** **:** **J Exp Clin Cancer Res 44, 185 (2025)**

**https://doi.org/10.1186/s13046-025-03453-0**

Following publication of the original article [1], the authors identified an error in the name of one of the authors. The given name and the family name were switched. The correct is presented below:

Current author name: Scarsella Marco

Correct author name: **Marco Scarsella**

Furthermore, errors were also found in the Fig. 2 of the published version, specifically panel E and panel F. The correct figure is presented below:

Incorrect Fig. 2

**Fig. 2:**
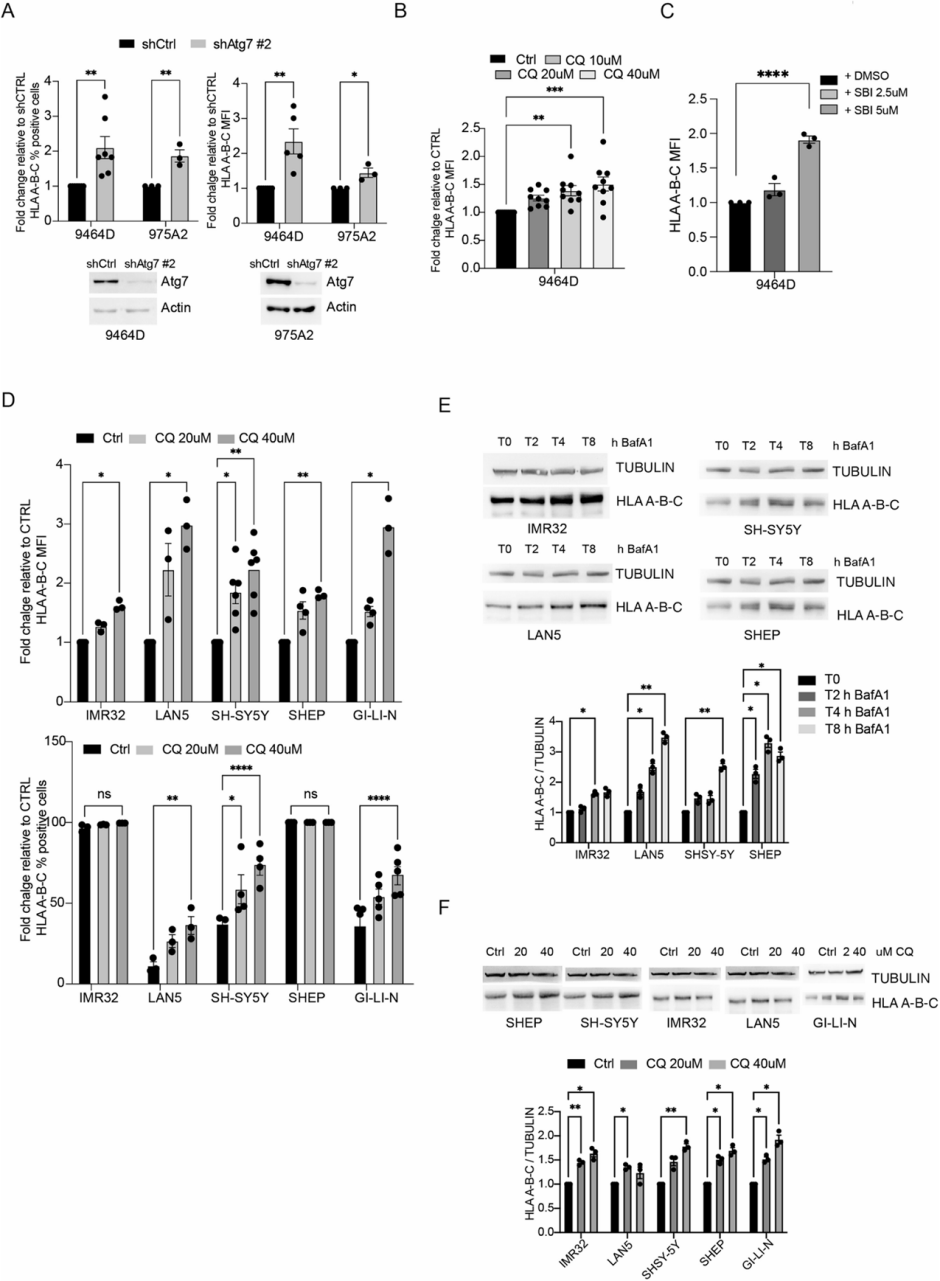
Autophagy inhibition stimulates MHC-I expression in NB. **A)** MHC-I levels were determined by flow cytometry in both 9464D and 975A2 cells after *Atg7* downregulation by lentiviral infection (shAtg7#2 or shCtrl). Data ± SEM are presented as percentage of positive cells (left) and mean fluorescence intensity (MFI) (right) normalized over shCtrl cells (unpaired Student’s t-test). Each dot represents an independent experiment. Representative immunoblotting shows Atg7 protein levels. Actin was used as a loading control; **B-C)** MHC-I expression was determined by flow cytometry after treatment with the indicated doses of CQ **(B)** and SBI-0206965 **(C)** respectively for 48 h in the 9464D cell line. Data ± SEM are presented as mean fluorescence intensity (MFI) normalized over shCtrl cells (one-way ANOVA followed by Tukey post hoc test); **D)** MHC-I expression was determined by flow cytometry in a panel of human NB cell lines after treatment with 20 or 40 µM CQ respectively. Data ± SEM are presented as MFI (top) and percentage of positive cells (bottom) normalized over shCTRL cells (one-way ANOVA); **E–F)** WB analysis of MHC-I protein levels after autophagy inhibition by Bafilomycin A1 (BafA1, 100 nM) or CQ in different human NB cell lines. Representative immunoblotting shows MHC-I expression levels. TUBULIN was used as a loading control. Densitometric analysis of MHC-I expression levels over TUBULIN is shown. Data ± SEM are presented and significance is calculated using two-way ANOVA (*p < 0.05, **p < 0.01; *n* = 3)

Correct Fig. 2

**Fig. 2:**
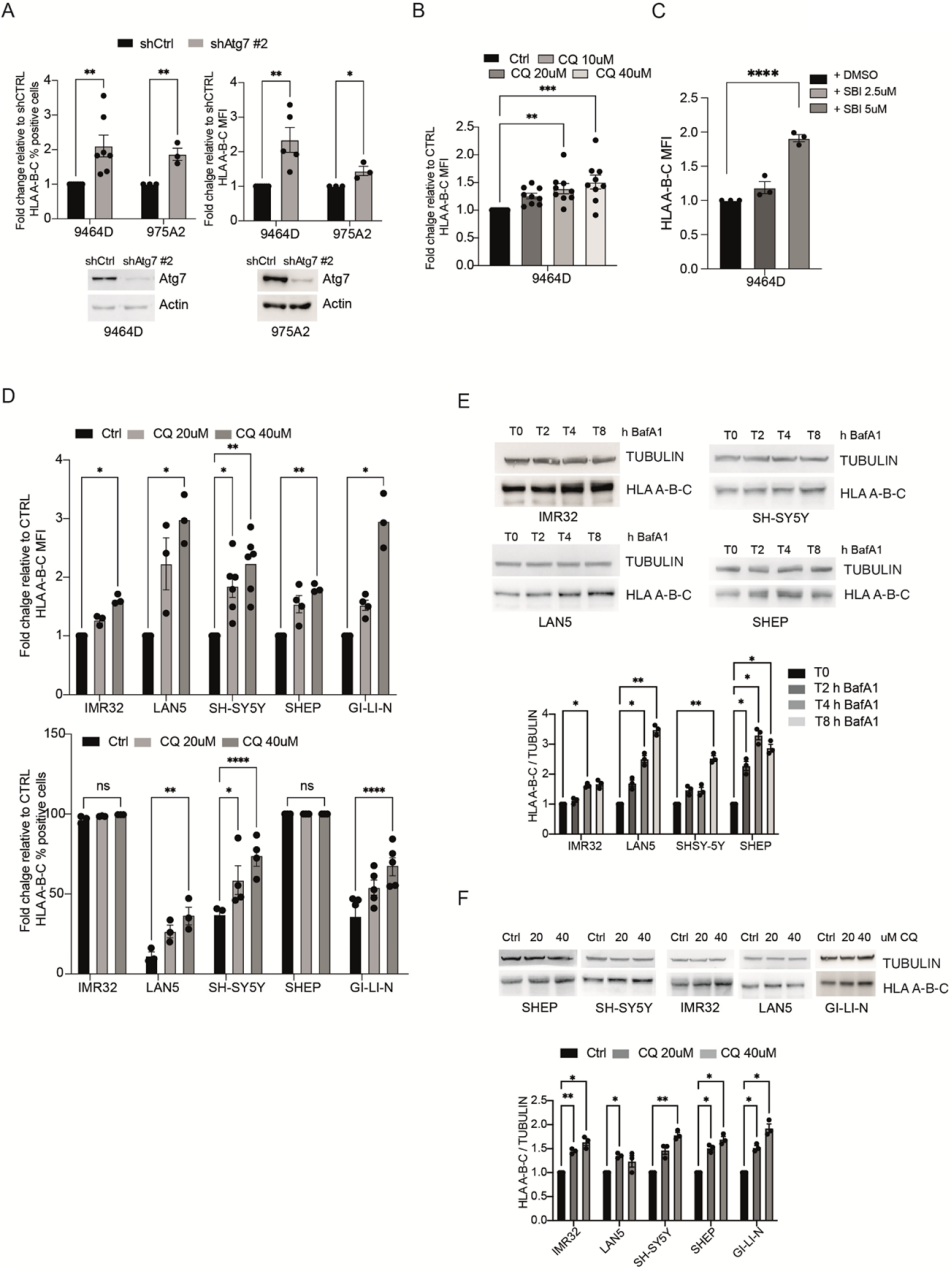
Autophagy inhibition stimulates MHC-I expression in NB. **A)** MHC-I levels were determined by flow cytometry in both 9464D and 975A2 cells after *Atg7* downregulation by lentiviral infection (shAtg7#2 or shCtrl). Data ± SEM are presented as percentage of positive cells (left) and mean fluorescence intensity (MFI) (right) normalized over shCtrl cells (unpaired Student’s t-test). Each dot represents an independent experiment. Representative immunoblotting shows Atg7 protein levels. Actin was used as a loading control; **B-C)** MHC-I expression was determined by flow cytometry after treatment with the indicated doses of CQ **(B)** and SBI-0206965 **(C)** respectively for 48 h in the 9464D cell line. Data ± SEM are presented as mean fluorescence intensity (MFI) normalized over shCtrl cells (one-way ANOVA followed by Tukey post hoc test); **D)** MHC-I expression was determined by flow cytometry in a panel of human NB cell lines after treatment with 20 or 40 µM CQ respectively. Data ± SEM are presented as MFI (top) and percentage of positive cells (bottom) normalized over shCTRL cells (one-way ANOVA); **E–F)** WB analysis of MHC-I protein levels after autophagy inhibition by Bafilomycin A1 (BafA1, 100 nM) or CQ in different human NB cell lines. Representative immunoblotting shows MHC-I expression levels. TUBULIN was used as a loading control. Densitometric analysis of MHC-I expression levels over TUBULIN is shown. Data ± SEM are presented and significance is calculated using two-way ANOVA (*p < 0.05, **p < 0.01; *n* = 3)

The correction does not compromise the validity of the conclusions and the overall content of the article. The author group has been updated above and the original article [1] has been corrected.
