# Supplementary material for: Inhibition of autophagy enhances the antitumor efficacy of T/CAR T cell against neuroblastoma
Source: J Exp Clin Cancer Res. 2025 Jul 3;44:185. doi: 10.1186/s13046-025-03453-0 (PMC12224479; doi:10.1186/s13046-025-03453-0)
Supplement: Supplementary file 1 — Supplementary Material 1 [file 13046_2025_3453_MOESM1_ESM.pdf]

Supplementary Figures Legends

Suppl. Figure 1. A) WB analysis of 9464D and 975A2 protein extracts after *Atg7* downregulation using two different shRNA (shAtg7#1, shAtg7#2) or with control (shCtrl) by lentiviral infection. Representative immunoblotting of Atg7, p62 and LC3 is shown. Actin was used as a loading control. Densitometric analysis of Atg7, LC3II and p62 expression levels over Actin is shown. Data ± SEM are presented, and significance is calculated using one-way ANOVA (*p< 0.1, **p< 0.01; n=3). **B)** Representative immunoblotting of Ulk1, pAtg14, Atg14, and LC3 in protein extracts of 9464D and 975A2 cell lines treated with the indicated concentrations of SBI-0206965 and CQ for 48 hours respectively. Densitometric analysis of p-Atg14 over Atg14/Actin is shown. Data ± SEM are presented, and significance is calculated using one-way ANOVA (***p< 0.01; n=3). **C)** Analysis of 3D growth of 9464D cells after *Atg7* downregulation by lentiviral infection (shAtg7#1 or shCtrl). The spheroid area was calculated at the indicated time points. Data are presented as mean ± SEM calculated over 24 hours (ns, not significant; two-way ANOVA, n=6). Representative images are shown. **D)** Representative images of 9464D spheroids treated with 40μM CQ and acquired at the indicated time points. **E)** Analysis of cell viability of both 9464D and 975A2 NB cell lines treated with the indicated doses of SBI-0206965 or CQ 24 hours after plating. Cells were analyzed by MTS assay at the indicated time points. Bar graphs show the 490 nm absorbance mean ± SEM calculated over control cells (*p< 0.1, **p< 0.01; two-way ANOVA, n=3). **F)** Analysis of cell viability of SH-SY5Y, LAN-5, and IMR32 cells treated with the indicated doses of SBI-0206965 by MTS assay as in E. Data are presented as the mean ± SEM calculated over control cells (*p< 0.1, **p< 0.01; two-way ANOVA n=3); **G**) Analysis of spheroid area of SH-SY5Y, LAN-5 and IMR32 cells after treatment with different concentration of SBI-0206965. The spheroid area was calculated at the indicated time points. Data are presented as mean ± SEM calculated over 24 hours (two-way ANOVA, n=3).

Supp. Figure 2. A) Representative immunoblotting of pAtg14 and Atg14 in protein extracts of 9464D cell line treated with the indicated concentrations of ULK-101 for 24 hours. Densitometric analysis of p-Atg14 over Atg14/Hsp90 is shown. Data ± SEM are presented, and significance is

calculated using one-way ANOVA (***$p < 0.01$; n=3). **B**) MHC-I expression was determined by flow cytometry after treatment with the indicated doses ULK-101 for 24 and 48 hours in the 9464D cell line. Data ± SEM are presented as mean fluorescence intensity (MFI) normalized over shCtrl cells (one-way ANOVA followed by Tukey post hoc test). (n=3).

Suppl. Figure 3. A) Volcano plot of mass-spectrometry identified proteins derived from ex vivo tumor tissue. Fold Change of shAtg7 versus shCtrl modulated proteins are represented (Red: upregulated, Blu: downregulated DEPs in shAtg7 tumors). **B)** Network analysis of shAtg7 versus shCtrl-derived tumors. Red: upregulated Blu: downregulated proteins, Size: -log p-value. **C**) Percentage of $CD69^+$ $PD1^+$ and $CD69^-$ $PD1^+$ cells of both $CD4^+$ and $CD8^+$ T cell populations collected from 9464D bearing C57BL/6 mice treated with CQ or vehicle respectively. **D-E)** Immunophenotype of infiltrating T cells ($CD45^+$, $CD3^+$, $CD4^+$, $CD8^+$ $CD44^+$/$CD62L^-$ effector memory (EM)) in shAtg7#2 and shCtrl 975A2 tumors evaluated by flow cytometry. The number of events was normalized on tumor weight. Data are presented as mean ± SEM calculated by Mann Whitney test. (n>10) *$p < 0.05$.

Supp. Figure 4. A) CD8 expression levels of sorted T cells (n=4). Representative dot plot for CD8 expression of $CD3^+$ population. **B**) Functional activity in long-term killing assays of PRAME-TCR-T cells against THP-1 and HL-60 cells in the E:T ratio of 1:2 (n=3).

Supp. Figure 5. A) Schematic representation of T-cells transduced with a third-generation CAR directed against $GD2^+$ expressed on NB cells. **B)** GD2.CAR expression levels on non-transduced (NT) control and GD2.CAR T cells (n=3). Representative dot plot of NT control cells and GD2.CAR transduced T cells. Cells were surface stained with 1A7 antibody (anti-GD2.CAR) with subsequent staining of PE-labeled secondary antibody. **C)** Functional activity in long-term killing assays of GD2.CAR T cells against SH-SY5Y in an E:T ratio of 1:2 (n=3). **D)** Cytotoxic effects of 5μM SBI-0206965 (SBI) and 40μM CQ treatment in CHLA-255-GFP^+ and SH-SY5Y-GFP^+ spheroids respectively. Data are presented as mean ± SEM calculated by two-way ANOVA (n=3 experiments). **(E-F)** Long-term killing assays of NT cells co-cultured at different E:T ratios

with 24 hours SBI-0206965 (5μM)-pretreated spheroids of CHLA-255-GFP$^+$ and SH-SY5Y-GFP$^+$ cells. Data are presented as mean ± SEM calculated by two-way ANOVA (n=3 experiments). **G)** Long-term killing assays of GD2.CAR T cells co-cultured at different E:T ratios with 24 hours SBI-0206965 (5μM)-pretreated spheroids of CHLA-255-GFP$^+$ and SH-SY5Y-GFP$^+$ cells respectively. Data are presented as mean ± SEM calculated by two-way ANOVA (n=3 experiments). *p< 0.05, **p< 0.01. **H)** Representative images and average (AVG) GFP intensity of 24 hours CQ-pretreated SH-SY5Y-GFP$^+$ spheroids co-cultured with GD2.CAR T cells at 1:3 E:T ratios and for the indicated time points. Data are presented as mean ± SEM calculated by two-way ANOVA (n=3 experiments). *p< 0.05, **p< 0.01. **I**) Average (AVG) GFP intensity of 24 hours CQ-pretreated CHLA-255-GFP$^+$ spheroids co-cultured with GD2.CAR T cells at 1:3 E:T ratios and for the indicated time points. Data are presented as mean ± SEM calculated by two-way ANOVA (n=3 experiments). *p< 0.05. **J)** Long-term killing assays of GD2.CAR T cells co-cultured at 1:3 and 1:2 of E:T ratios with shAtg7 *vs* shCTRL SH-SY5Y-GFP$^+$ spheroids. Data are presented as mean ± SEM calculated by two-way ANOVA (n=3 experiments). *p< 0.05, **p< 0.01, ***p< 0.001.

Supp. Figure 6. A) Tumor growth analyses in mice receiving NT cells +PBS or combined with CQ. Statistical analyses by two-way ANOVA followed by Šídák's multiple comparisons test. **B)** Flow cytometry analyses of tumor infiltrated cells (GFP$^+$) after spleen dissociation of mice receiving GD2.CAR T or NT cells and CQ or PBS respectively. Data are presented as mean ± SEM. Statistical analysis by one-way ANOVA. **(C)** Representative gate strategy of CD4$^+$, CD8$^+$ (both gated on CD3$^+$ cells), CD95$^+$ and HLA-DR$^+$ T cells (both gated on CD4$^+$ or CD8$^+$ cells) in CD45$^+$/FITC$^-$ spleen infiltrating cells. **(D)** Representative gate strategy of GD2.CAR T CD4$^+$, CD8$^+$ (both gated on CD3$^+$ cells), and HLA-DR$^+$ T cells (both gated on CD4$^+$ or CD8$^+$ cells) in CD45+/FITC- spleen infiltrating cells. **E-F)** Percentage of CAR T cells, CD4$^+$, CD8$^+$, and HLA-DR$^+$ cells gated on GD2.CAR T cells isolated from spleens of PBS or CQ-receiving mice. Spleens were collected at the final time point and flow cytometry analyses were performed. Statistical analysis by unpaired Student's t-test. *p< 0.05, **p< 0.01. **G-H-I**) Frequency of positive CD4 and CD8 T cells, frequency of positive KI67 and HLA-DR in both CD4$^+$ and CD8$^+$ cells, frequency of positive PD1, Lag3 or TIM3 in both CD4 and CD8 T cells. Statistical analysis

by unpaired Student's t-test. **J**) Frequency of positive PD1, Lag3 and TIM3 in both $CD4^+$ and $CD8^+$ T cells. Statistical analysis by unpaired Student's t-test.
