# Supplementary figures and images for: Inhibition of autophagy enhances the antitumor efficacy of T/CAR T cell against neuroblastoma
Source: J Exp Clin Cancer Res. 2025 Jul 3;44:185. doi: 10.1186/s13046-025-03453-0 (PMC12224479; doi:10.1186/s13046-025-03453-0)

Supp. Figure 1

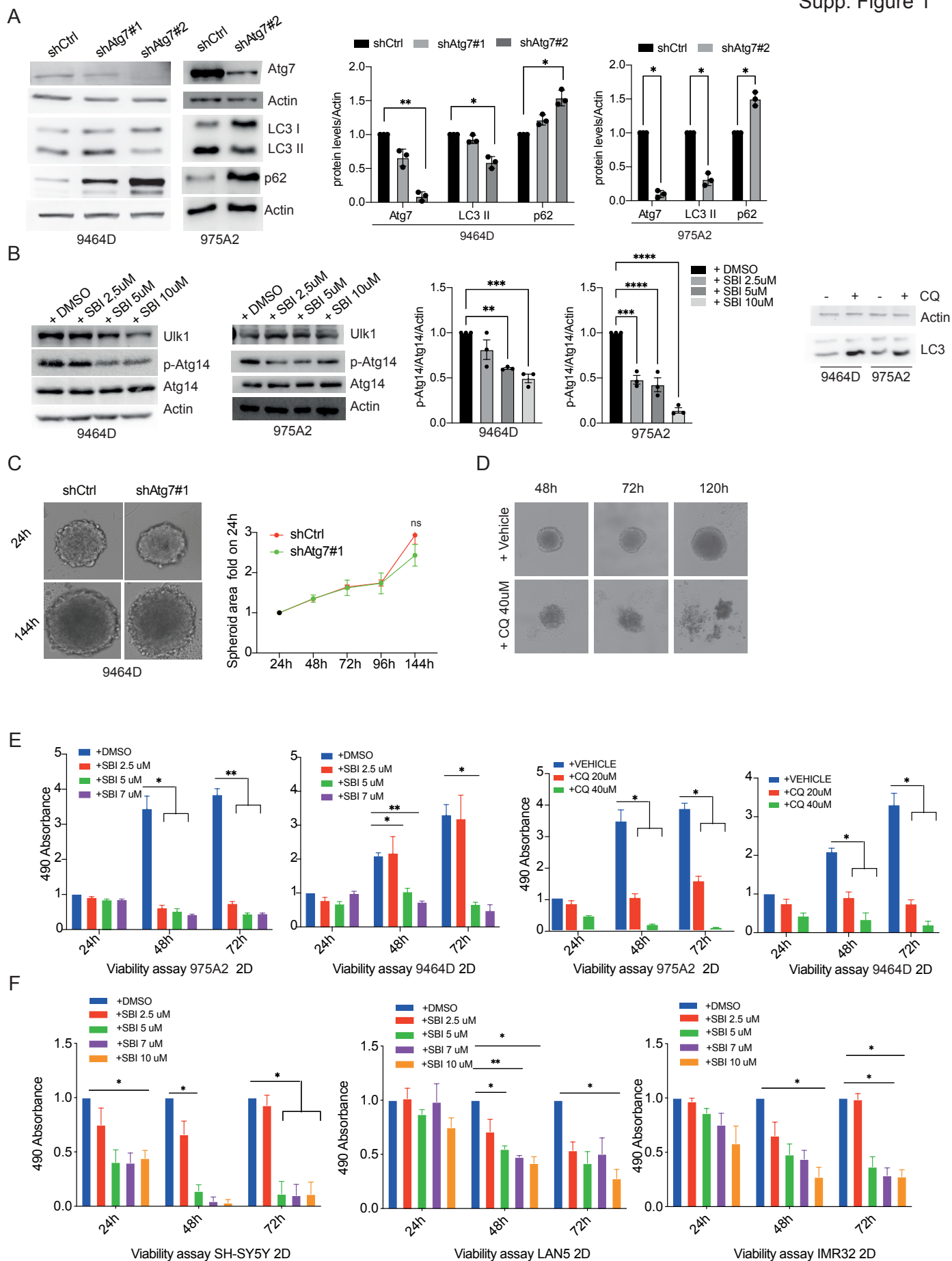

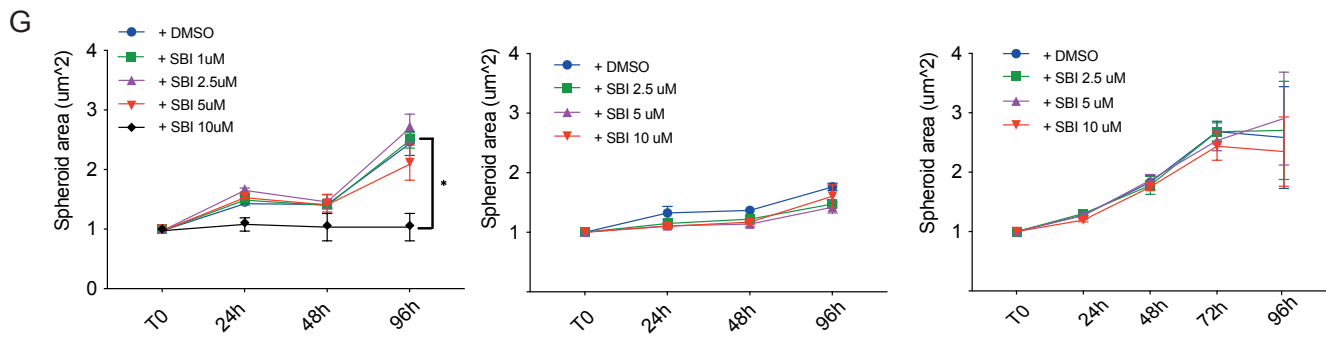

A

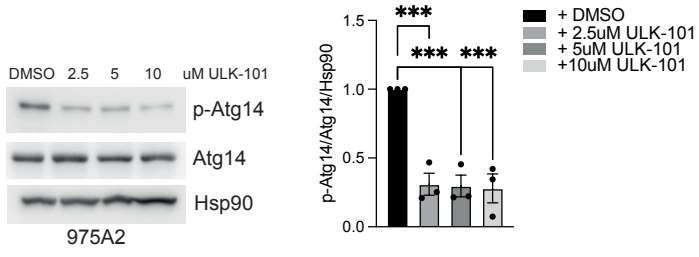

B

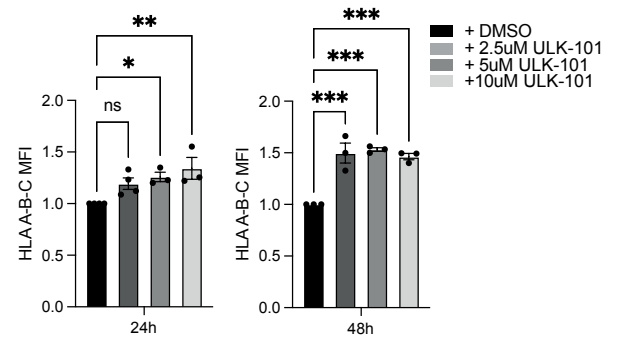

Supp Figure 3

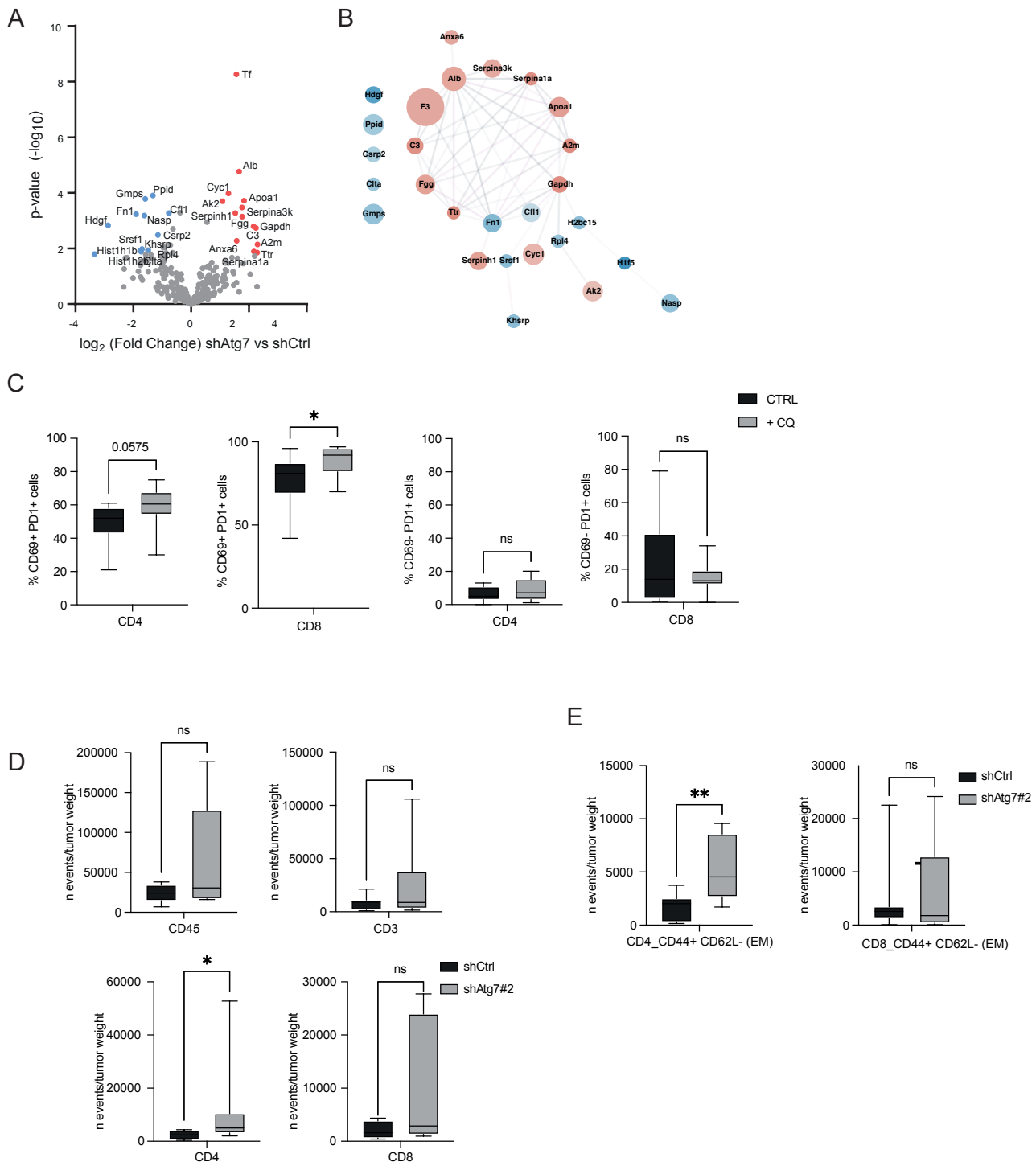

Supp Figure 4

A

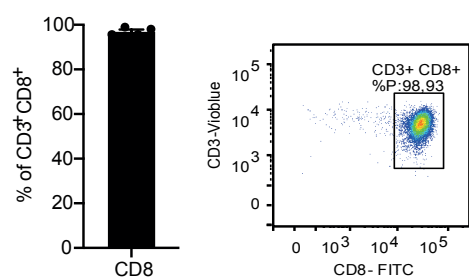

B

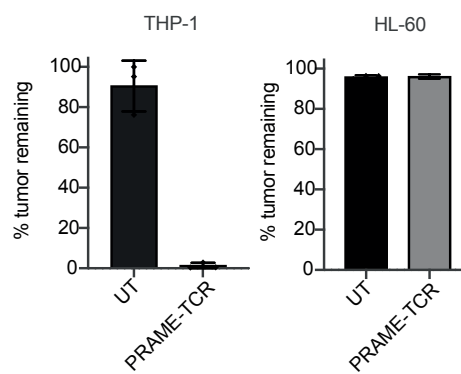

Supp. Figure 5

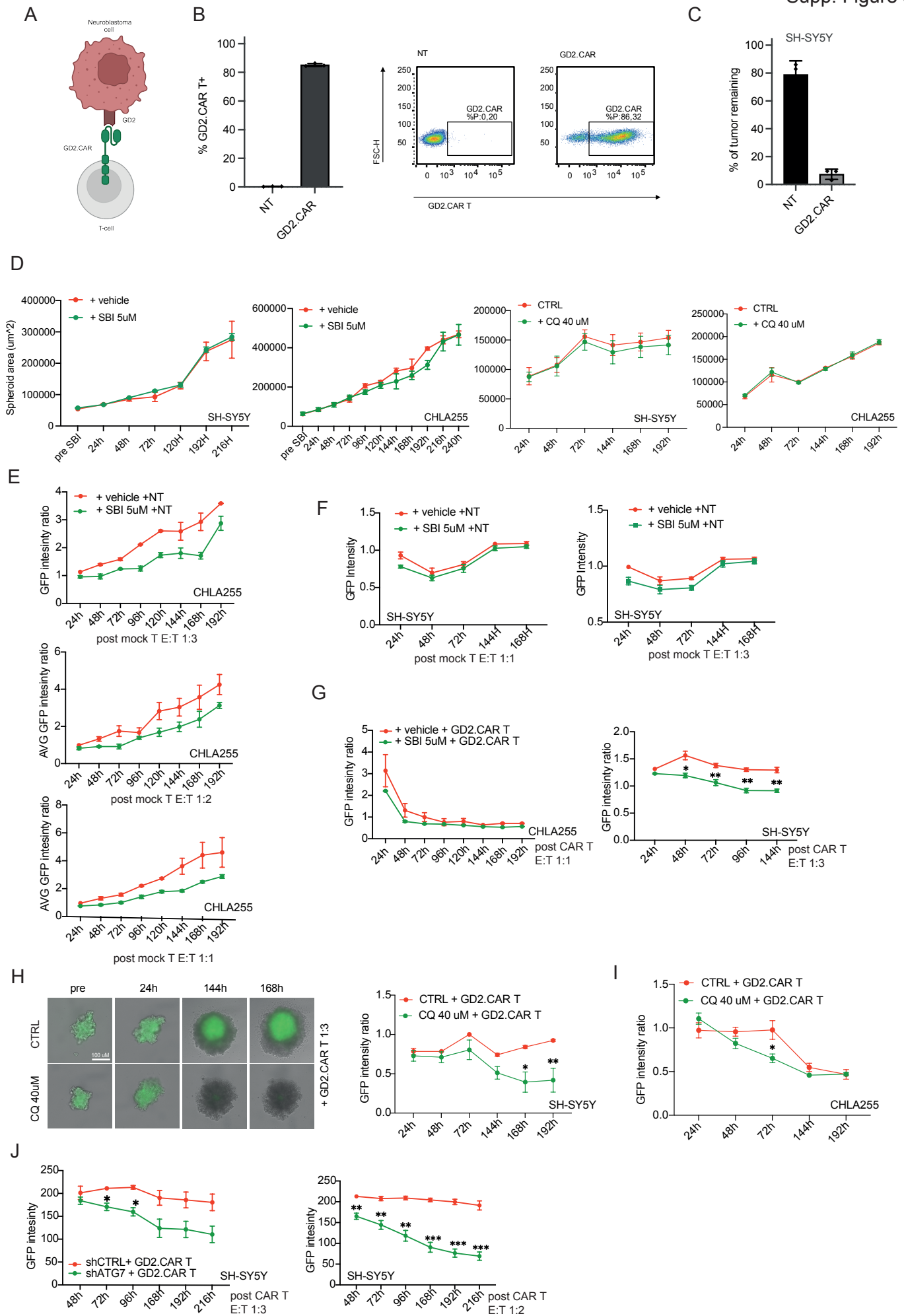

Supp. Figure 6

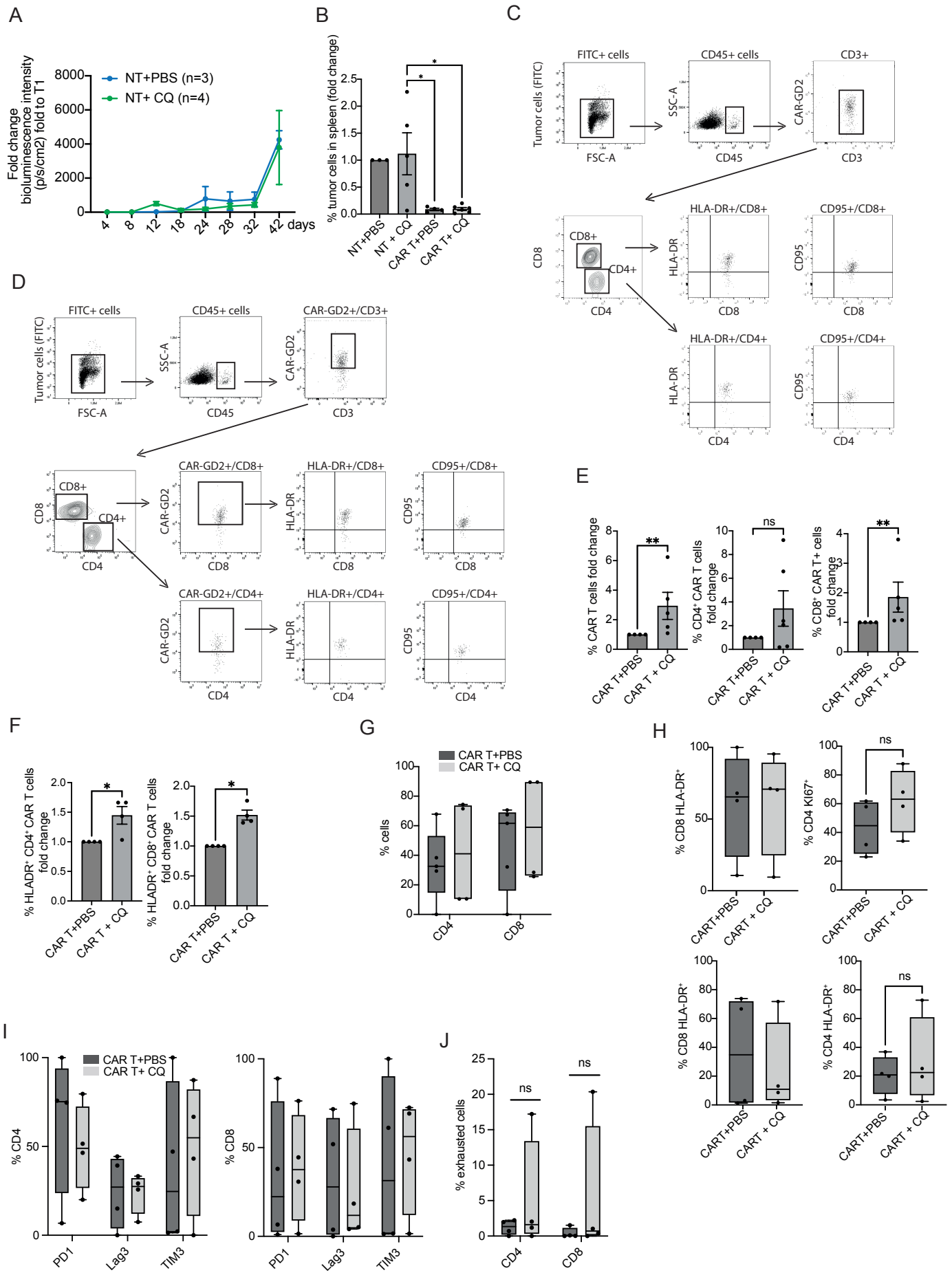

Supplement: Supplementary file 2 — Supplementary Material 2 [file 13046_2025_3453_MOESM2_ESM.pdf]
