# Supplementary material for: Inhibition of autophagy enhances the antitumor efficacy of T/CAR T cell against neuroblastoma
Source: J Exp Clin Cancer Res. 2025 Jul 3;44:185. doi: 10.1186/s13046-025-03453-0 (PMC12224479; doi:10.1186/s13046-025-03453-0)
Supplement: Supplementary file 3 — Supplementary Material 3 [file 13046_2025_3453_MOESM3_ESM.docx]

**Table S1**

| **Antibodies: Flow Cytometry** | **Cat Number, Source** |
| --- | --- |
| **CD45** - FITC ANTI-MOUSE | 553079, BD Biosciences |
| **CD3** - BUV395 ANTI-MOUSE | 563565, BD Biosciences |
| **CD4** - BV605 ANTI-MOUSE | 743156, BD Biosciences |
| **CD8A** - BUV805 ANTI-MOUSE | 612898, BD Biosciences |
| **CD62L** - BV421 ANTI-MOUSE | 562910, BD Biosciences |
| **CD44** - BV786 ANTI-MOUSE | 563736, BD Biosciences |
| **CD69** - APC-Cy7 ANTI-MOUSE | 561240, BD Biosciences |
| **H-2Kb/H-2Db (MHC I**) - PE ANTI-MOUSE | 114607, Biolegend |
| **CD274 -** BUV737 ANTI-MOUSE | 741877 BD Biosciences |
| **CD3 –** Vioblue ANTI-HUMAN | 130-113-133, Miltenyi Biotec |
| **CD8** – FITC ANTI-HUMAN | 130-113-157, Miltenyi Biotec |
| **CD45** - KrOR ANTI-HUMAN | B36294, Beckman Coulter |
| **CD3** - APC ANTI-HUMAN | 130-113-135, Miltenyi |
| **CD4** - BV650 ANTI-HUMAN | 563875, BD Biosciences |
| **CD8** - Pc7 ANTI-HUMAN | 335822, BD Biosciences |
| **1A7 clone Antibody (ANTI GD2)** | Homemade (34) |
| **PE conjugated ANTI-mouse Kappa** (secondary Ab) | 349073, BD Biosciences |
| **CD95** – BV421 ANTI-HUMAN | 562616, BD Biosciences |
| **HLA-DR** – BV786 ANTI-HUMAN | C78087, Beckman Coulter |
| **HLA A-B-C** APC ANTI-HUMAN | 555555, BD Biosciences |
|  | |
| **Antibodies: MACSima^TM^ Imaging Cycling** | **Source** |
| **Ki67, CD3, CD4, CD8, CD44, CD45RA, CD45RO, CD57, CD183, CD196, LAG3 (CD223), PD-1 (CD279), TIM3 (CD366), FoxP3, HLA-DR** | Miltenyi Biotec |
